# BRG1 Loss Is Frequent in Lung Cancer and Transforms Lung Epithelial Cells via Transcriptional and Epigenetic Reprograming

**DOI:** 10.3390/cancers17183092

**Published:** 2025-09-22

**Authors:** Mathewos Tessema, Christin M. Yingling, Loryn M. Phillips, Kieu Do, Maria A. Picchi, Randy Willink, Steven A. Belinsky

**Affiliations:** Lung Cancer Program, Lovelace Biomedical Research Institute, Albuquerque, NM 87108, USA

**Keywords:** BRM, SMARCA2, SMARCA4, SWI/SNF, BAF, pre-malignancy, smoking

## Abstract

Background/Objectives: The BRG1 loss-of-function (LOF) mutation is found in ~10% of non-small cell lung cancer (NSCLC) cases, but its role in lung tumorigenesis is unclear and so it is investigated in this study. Methods: BRG1-knockout (KO) lines were generated from various non-malignant, pre-malignant, and malignant human lung epithelium-derived cell lines using CRISPR. The effects of BRG1-KO on cell growth, the transcriptome, the methylome, and epigenetic therapy were compared with those of wild-type (BRG1-WT) isogenic controls using standard in vitro and in vivo assays. Results: The BRG1 protein was expressed in all non-/pre-malignant lung epithelial cells but lost in 47% (14/30) of NSCLC cell lines. BRG1-KO and cigarette smoke (CS) exposure individually transformed human bronchial epithelial cell lines (HBECs), as evidenced by anchorage-independent growth. BRG1-KO and CS produced additive to synergistic effects on sensitivity to transformation that differed across HBECs. RNA-seq analysis revealed that BRG1-KO significantly changed the expression of over 8500 genes on average, impacting lung development, function, damage repair, and cancer pathways, including axonal guidance, pulmonary wound healing, and epithelial-to-mesenchymal transition (EMT). BRG1-KO also led to the hypermethylation of >47,000 promoter CpGs within ~9500 genes on average in different HBECs, including silencing of epithelial genes involved in EMT and tumor suppressor genes. BRG1-KO also moderately increased the in vitro and in vivo sensitivity of NSCLC cells to some epigenetic drugs. Conclusions: BRG1-LOF is frequent in NSCLC; can drive the transformation of lung epithelial cells such that they acquire properties of pre-malignant cells, indicating a potential role in lung cancer initiation; and sensitizes lung tumors to epigenetic therapy.

## Simple Summary

BRG1 protein expression or its normal functions are lost in many cancers, including ~10% of lung cancer cases, due to mutation. However, how these losses contribute to the development of lung cancer is not clearly known. This study used human lung epithelial cell-derived normal, pre-cancer, and cancer cell lines to investigate the initiation and progression of lung cancer. Our results show that BRG1 loss induces epigenetic (non-genetic) and expression changes in thousands of genes genome-wide that lead to the early transformation of normal cells into pre-cancer cells. In established cancer cells, BRG1 loss and its downstream epigenetic and gene expression changes make the cancer cells become sensitive to epigenetic drugs. Taken together, these results indicate that BRG1 loss contributes to early lung cancer development and could be exploited to improve the treatment of this deadly disease.

## 1. Introduction

Personalized medicine targeting cancer-driver mutations has improved cancer therapy. Non-small cell lung cancer (NSCLC) patients, who account for over 85% of all lung cancer cases, are among those who have benefited the most from these advances [1]. Molecular testing to identify and preselect patients displaying these targetable mutations has now become a critical component of the standard-of-care management of NSCLC [1]. However, the success of this approach has so far been limited to inhibition of aberrantly activated oncogenes, mainly the tyrosine kinase families, such as EGFR; BRAF; ALK; and, more recently, the G12C mutation of KRAS [1,2]. This limitation has left many important and highly prevalent cancer-promoting loss-of-function (LOF) mutations, such as TP53, LKB1, and many others, therapeutically unexploited. The primary reason for this exclusion is the technical challenges in directly targeting these so-called ‘undruggable’ mutations via gene or drug delivery primarily to cancer cells without significant side effects. A potentially promising alternative method to selectively treat these kinds of cancers is to identify and target synthetic lethality or unique druggable vulnerabilities created by or strongly linked to a specific LOF mutation without directly engaging the LOF itself [3]. Targeting of the SMARCA4 LOF mutation found in NSCLC and many other cancers serves as an excellent example of the potential of this approach [4,5,6,7,8].

SMARCA4 (Brahma-related gene-1, BRG1) and its paralog SMARCA2 (Brahma, BRM) encode the two mutually exclusive ATPase subunits of mammalian SWI/SNF (SWItch/Sucrose Non-Fermentable), a 10–15-subunit complex. This master chromatin-remodeling complex uses the energy from the ATPase activity of BRG1/BRM to move or evict nucleosomes, open the chromatin, and thereby control the access to DNA and regulate vital cellular processes, such as DNA replication, repair, and gene expression [9,10,11]. However, these critical roles have also made SWI/SNF one of the most frequent targets in cancer; its subunits, including BRG1, are mutated in ~20% of all cancers, often resulting in LOF of the specific protein [9,10,12]. Germline or somatic BRG1-LOF mutations are characteristic and diagnostic features in nearly 100% of highly aggressive and poorly differentiated tumors with a rhabdoid morphology, such as small cell carcinoma of the ovary hypercalcemic type (SCCOHT) and SMARCA4-deficient thoracic sarcomas (SMARCA4-DTS) [6,7]. Similarly, LOF mutations in BRG1 or SMARCB1 (another SWI/SNF subunit) respectively drive ~5 and 95% of malignant rhabdoid tumors (MRTs) and serve as key diagnostic markers for these highly aggressive childhood cancers [5]. We and others have shown that the BRG1-LOF mutation is also found in ~10% of NSCLCs of smokers and never-smokers [4,8,13,14].

Previous studies have shown that BRG1-LOF promotes malignancy and sensitizes cancer cells to various drugs [15,16,17]. As the ATPase activity of BRG1 is also vital to protecting and actively reversing epigenetic silencing of genes by polycomb repressor complexes (PRCs), its LOF appears to drive cancer via major epigenetic and transcriptional reprograming [18,19]. An increasing number of studies have shown that BRG1 loss promotes more aggressive lung tumors [20,21,22] and creates therapeutic vulnerabilities that could be targeted for more effective treatment [23,24,25]. Preclinical studies show the increased sensitivity of BRG1-LOF tumors to many types of epigenetic drugs targeting histone deacetylases, PRCs, and bromodomain proteins [17,23,24]. The increased dependency of BRG1-deficient tumors on the only remaining alternative ATPase subunit SMARCA2 and/or other compensatory survival pathways also make them sensitive to targeted inhibition of these processes [23,26,27,28]. The increased sensitivity of BRG1-LOF tumors to degraders or inhibitors of SMARCA2, aurora kinase, CDK4/6, oxidative phosphorylation, and other survival factors demonstrates the broad therapeutic vulnerabilities of these cancers [25,26,27,28,29,30]. These findings, along with the availability of well-optimized BRG1 mutation and expression (immunohistochemistry) assays in the clinic [31], underscore the importance and promise of accurately identifying and targeting BRG1-deficient lung tumors for personalized therapy.

The goal of this study was to develop well-controlled BRG1-deficient lung cancer models and to study their roles at various stages of NSCLC development. CRISPR was used to knockout (KO) BRG1 in non-malignant, pre-malignant (cigarette smoke-transformed), and malignant (NSCLC) human lung epithelium-derived cell lines, and the effects on their early initiation, progression, and therapeutic vulnerability were investigated in vitro and in vivo. The genome-wide impacts of BRG1-LOF on gene expression, promoter CpG methylation, and the top significantly affected signaling pathways were systematically evaluated using multiple BRG1-KO and wild-type (WT) isogenic control lines. The relationships between BRG1-KO-induced epigenetic and expression changes were determined and the direction of the changes in comparison to NSCLC were demonstrated using selected commonly observed abnormalities in lung cancer. Finally, the impact of BRG1-LOF and its broad epigenome reprograming effects on therapeutic vulnerabilities of NSCLC to a library of epigenetic drugs were investigated in vitro, and the most promising drug combinations were tested in vivo.

## 2. Materials and Methods

### 2.1. Cell Lines

A total of 47 cell lines (Table S1) derived from human lung epithelial cells or NSCLC tumors were evaluated. These included hTERT/cdk4 immortalized human bronchial epithelial cell lines (HBECs, *n* = 7), small airway epithelial cell lines (HSAECs, *n* = 5), cigarette smoke (CS)-transformed HBEC/HSAEC lines (CST, *n* = 5) generated through chronic in vitro CS aerosol exposure as previously described [32], and commonly used NSCLC cell lines (*n* = 30). The HBECs and HSAECs were obtained from Drs. Shay and Minna, Southwestern Medical Center, Dallas, TX, and cultured as previously described [33,34]. The NSCLC cell lines were obtained from, authenticated by, and cultured as recommended by the American Type Culture Collection (Manassas, VA, USA). All cell line-based experiments start with authenticated and mycoplasma contamination-free cell lines that were passed for a maximum of 6 months post-resuscitation.

### 2.2. BRG1 Knockdown, Knockout, and Western Blotting

BRG1 was transiently knocked down using in vitro transfection of cells with BRG1-specific siRNA (siBRG1 and s13141) or scrambled control siRNA (siCont, cat. no. 4390843) obtained from ThermoFisher Scientific (Waltham, MA, USA) as previously described [35]. BRG1 was stably knocked out using a combination of the Brg-1 CRISPR/Cas9 knockout (KO) Plasmid (sc-400168) and the Brg-1 HDR Plasmid (sc-400168-HDR) or a non-targeting Control CRISPR/Cas9 Plasmid (sc-418922), all obtained from Santa Cruz Biotechnology Inc. (Dallas, TX, USA), as described by the manufacturer. BRG1-KO clones were selected with puromycin resistance and Red Fluorescent Protein expression and expanded as needed. Protein expression was detected using 50 μg total protein and standard Western blot assays with the following antibodies, all obtained from Santa Cruz. Blots were blocked with 5% milk in BST, stained with primary anti-BRG1 (sc-17796) at 1:1000 or anti-β-actin (sc-47778) at 1:5000 and horseradish peroxidase (HRP)-conjugated mouse IgG Fc binding protein (m-IgG Fc BP) secondary (sc-525409) at 1:1000 dilutions, and images were visualized using the Bio-Rad Image Lab 6.1 software.

### 2.3. Gene Expression

RNA-sequencing and analysis were conducted at the genomics core facility of the University of New Mexico Comprehensive Cancer Center as previously described [36,37]. Briefly, cells were harvested in Tryzol, total RNA was isolated using the RNeasy Mini Kit obtained from Qiagen (Germantown, MD, USA), RNA integrity was assessed with an Agilent 2100 Bioanalyzer and an RNA Integrity Number extracted from the electropherogram to determine quality. RNA was sent to Azenta for library preparation, and sequencing libraries with PolyA selection were prepared and sequenced in 150 bp paired-end runs. A target depth of 30 million reads per sample was obtained using an Illumina HiSeq (Azenta San Diego, CA, USA). RNA-seq and DNA methylation data were generated (see the next section) and have been deposited in the NCBI database (GEO accession number: GSE307857). For validation of gene-specific expression, 1 µg total RNA was reverse transcribed, and RT-qPCR analyses of the target gene multiplexed with the endogenous control β-Actin were determined in triplicate using inventoried TaqMan assays (Life Technologies Pleasanton, CA, USA) as previously described [32,38].

### 2.4. DNA Methylation

Genomic DNA was extracted and 1.0 µg DNA bisulfite was modified using the EZ DNA methylation Gold Kit (Zymo Research Irvine, CA, USA) as previously described [35,39]. The modified DNA was amplified, enzymatically fragmented, precipitated, re-suspended in hybridization buffer, hybridized to the Illumina Infinium MethylationEPICv2.0 Beadchip (EPICv2.0), and run on an Illumina iScan System (Illumina, CA, USA) using Illumina’s standard protocol. Idat files were exported from Genome Studio (Illumina) and preprocessed with dye-bias normalization using the minfi package in R (3.3.2) to generate β-values for 935,000 probes with a detection *p*-value < 0.01 as previously described [40].

### 2.5. In Vitro Cell Survival, Migration, and Anchorage-Independent Growth

The effects of BRG1-KO and/or library 152-epigenetic drugs (item no. 11076) obtained from Cayman Chemical (Ann Arbor, MI, USA) on in vitro cell proliferation or survival were determined using MTT assays as previously described [41]. Briefly, BRG1-KO or WT isogenic control cells were seeded in 96-well plates at 1 × 10^4^ to 10^5^ cells/well based on the proliferation rate of the cell line and incubated for 4 days with or without specific treatments, and the cell survival rate was quantified with MTT. For cell migration, cells were serum-starved for 48 h and trans-well migration was measured using the CytoSelect 24-Well Cell Migration Assay (8 mm; Colorimetric) kit (Cell Biolabs San Diego, CA, USA) according to the protocol. Anchorage-independent growth was evaluated using soft agar, cultures were photographed, and colonies were counted using ImageJ 1.50i software as previously described [32].

### 2.6. In Vivo Tumor Growth and Drug Sensitivity

All in vivo procedures in this study were conducted under a Lovelace Biomedical Institutional Animal Care and Use Committee (IACUC)-approved protocol. The tumorigenicity of BRG1-KO and/or CS-transformed HBECs and the in vivo drug sensitivity of BRG1-KO or WT NSCLC (Calu6)-derived tumors were investigated using Crl:NU(NCr)-Foxn1nu mice (Athymic nude mice) obtained from Charles River as standard subcutaneous xenograft models, as previously described [41,42]. Briefly, 5 × 10^6^ transformed HBECs expanded from soft agar colonies (for tumorigenicity) or 2.5 × 10^6^ BRG1-KO or WT Calu6 cells (for drug sensitivity) were mixed 1:1 with Cultrex BME Type-3 extracellular matrix obtained from R&D Systems (Minneapolis, MN, USA) and subcutaneously injected on both sides of the dorsal abdomen in 6 male mice/group, and tumor growth was examined for up to 20 weeks (HBECs) or 8 weeks (Calu6) post-injection. After one week of BRG1-KO or WT Calu6-derived tumor growth, the mice bearing each tumor type (10–12 tumors/type) were treated 3 times/week for six consecutive weeks with intraperitoneal (i.p) injection of 0.5% (*v*/*v*) DMSO in DPBS (vehicle) or a combination of 4 mg/kg Panobinostat (LBH589) and 2 mg/kg 5-Azacytidine (5-Aza) (LBH + AZA). Tumor growth and body weight were measured twice/week until the end of the study 7 days post-final (6th weekly) treatment or earlier if tumor volumes exceeded 2000 mm^3^.

### 2.7. Data Analysis

RNA-seq data were analyzed using Illumina BaseSpace v1.6.0, adapter sequences were trimmed using DRAGEN FASTQ Toolkit app v1.3.1 in BaseSpace, and sequencing reads were aligned to human reference genome hg38 and quantified using Illumina’s DRAGEN RNA app v4.0.4. The count-based statistical method DESeq2 in Illumina’s DRAGEN Differential Expression app v4.2.4 was used to identify differences (log2 fold changes) between treatments (KO and CST) relative to controls. Wald-test *p*-values for significance testing were adjusted for multiple comparisons using the Benjamini–Hochberg method to determine the false discovery rate (FDR), and an FDR < 0.05 was used as the cut-off for identifying differentially expressed genes (DEGs) for each comparison. Statistically over-represented pathways within the DEGs were identified using Ingenuity Pathway Analysis software. Gene-specific RT-qPCR (Taqman) data were analyzed using the 2^(−ΔΔCt)^ relative expression method as previously described [32,38,43]. For EPICv2.0 DNA methylation data analysis, the average signal intensity between methylated and unmethylated probes was determined, β-values were calculated as previously described [40], and probes with ≥20% methylation changes between BRG1-KO vs. WT controls were considered differentially methylated probes (DMPs). A hypermethylated gene (HMG) is defined as a gene with ≥3 or ≥20% of all interrogated promoter probes showing ≥20% increased methylation. Statistical analyses were conducted in SAS 9.4 and R 3.3.2.

## 3. Results

### 3.1. BRG1 Loss of Function Is Frequent in Lung Cancer

**Figure 1:**
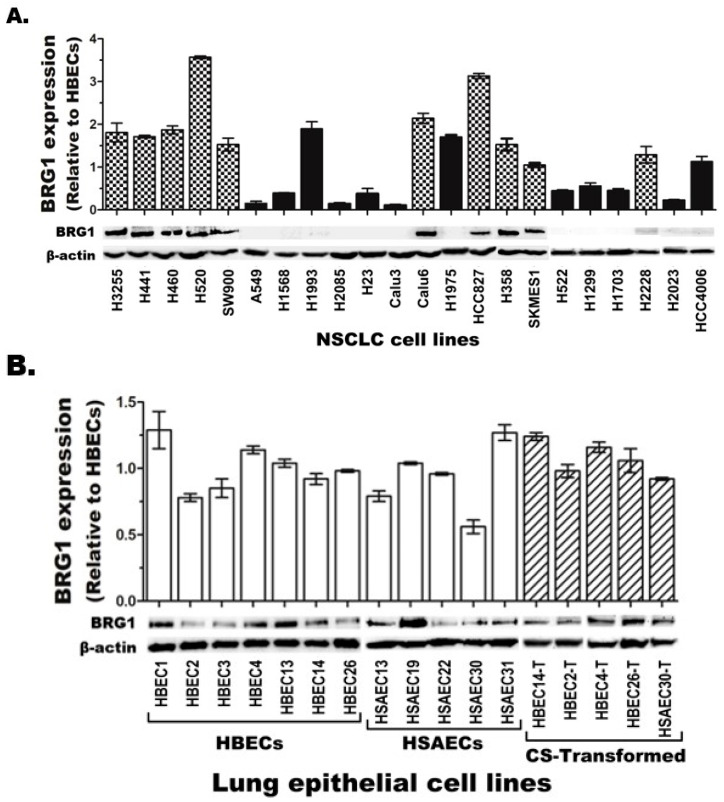
**BRG1 mRNA and protein expression in lung epithelium-derived cell lines.** (**A**) BRG1 mRNA expression (bar graph, top) in NSCLC cell lines showed major variation, while its protein (Western blots, bottom) was completely lost or barely detectable in nearly half of the cell lines evaluated. (**B**) In contrast, all normal and transformed lung epithelial cell lines showed comparable ranges of BRG1 mRNA and protein expression.

BRG1 mutation leading to its loss of protein expression or LOF is found in ~10% of cases of NSCLC and is a key diagnostic defect in ~100% of cases of SMARCA4-DTS, a thoracic cancer [4,7,8,44]. Both of these tumors are strongly associated with smoking, and NSCLC develops through the transformation of lung epithelial cells mainly by cigarette smoke (CS) [7,45]. Thus, this study first screened BRG1 mRNA and protein levels in 47 human lung epithelium-derived cell lines at different stages of malignancy (Supplementary Table S1). Immortalized human bronchial epithelial cell lines (HBECs, *n* = 7) and small airway epithelial cell lines (HSAECs, *n* = 5) were used to define normal BRG1 expression in human lung epithelia. These cell lines were selected because of their validated similarity to primary lung epithelial cells [46], while the long cell culture needed to generate and study BRG1-knockout (BRG1-KO) and/or CS-transformed (CST) lines in vitro without their undergoing senescence like normal primary lung epithelial cells was also allowed. The CST lines (*n* = 5) were generated via chronic (12-week) CS exposure of HBECs or HSAECs using our established protocol [32], and CST clones were selected based on their newly acquired anchorage-independent growth in soft agar, a hallmark of transformed cells [47]. The malignant NSCLC cell lines (*n* = 30) were established from tumors of different lung cancer patients. BRG1 mRNA and protein expression in each cell line was detected using gene-specific qPCR (TaqMan) and Western blot assays, using β-actin as an endogenous control. Although BRG1 mRNA was detected in all 47 cell lines evaluated, the level of expression in the NSCLC lines showed a wider variation range of 0.11–3.75-fold (Figure 1A, top panel, bar graph) compared to the relatively uniform expression (0.56–1.29-fold) in the immortalized and CST HBEC/HSAEC lines (Figure 1B, top panel). In contrast, BRG1 protein was barely or not detectable in 47% (14/30) of NSCLC cell lines (Figure 1A, bottom panel, Western blots) compared to BRG1 protein expression in all immortalized and CST HBECs and HSAECs (Figure 1B, bottom panel). The NSCLC cell lines that expressed little or no BRG1 protein mostly had significantly lower mRNA levels compared to the BRG1 protein-expressing lines (Figure 1A, black vs. dotted bars). While the BRG1 protein was mostly lost in the BRG1-mutant NSCLC cell lines (e.g., A549, H23, and H522), it was also seen in some BRG1-WT lines (e.g., Calu3; Table S1), likely due to non-genetic (epigenetic) silencing, as previously reported [25,48,49].

### 3.2. BRG1 Loss Transforms Immortalized Lung Epithelial Cells

**Figure 2:**
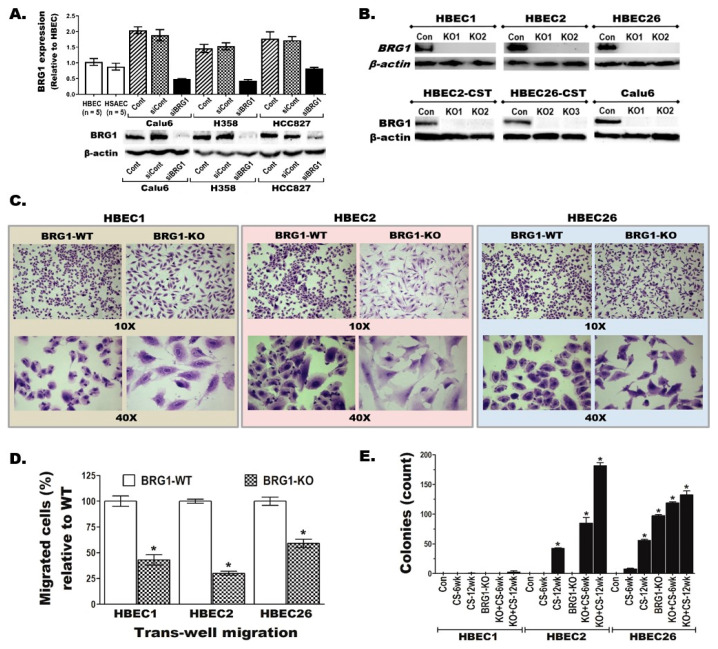
**BRG1 knockout transforms normal lung epithelial cell lines.** (**A**,**B**) BRG1 expression was (**A**) transiently knocked down (KD) using BRG1-specific siRNA or (**B**) stably knocked out (KO) using CRISPR-Cas9, and the reduced mRNA (bar graph) and reduced or complete loss of BRG1 protein expression (Western blots) is shown. (**C**–**E**) Loss of BRG1 in the BRG1-KO HBECs led to major changes in the (**C**) morphology, (**D**) trans-well migration, and (**E**) anchorage-independent growth capability in soft agar, indicating transformation of these HBECs into pre-malignant cells. The histology pictures in (**C**) were taken with the NanoZoomer^®^ Slide Scanner System, and the images shown are 10× and 40× magnifications. * *p* < 0.05.

Clearly elucidating whether BRG1-LOF plays a role in early lung cancer initiation requires well-controlled isogenic models. Thus, BRG1 was transiently knocked down (KD) or stably knocked out (KO) in HBECs using BRG1-specific siRNA (siBRG1) or CRISPR-Cas9, respectively. Compared to the corresponding parental or scrambled siRNA-transfected (siCont) controls, siBRG1 significantly reduced BRG1 mRNA and protein levels (Figure 2A and Figure S1A). Similarly, the BRG1 protein detected in the parental or CRISPR control cell lines was completely lost in the corresponding BRG1-KO lines (Figure 2B). Although these transient or stable changes did not significantly alter cell proliferation and/or survival, BRG1-KO induced major cell morphology, migration, and anoikis-resistant (anchorage-independent) growth changes in HBECs (Figure 2C–E and Figure S1B,C). The BRG1-KO-induced transition from the normal epithelial to elongated ‘fibroblast-like’ morphology (Figure 2C) and the significant reduction in trans-well migration (Figure 2D and Figure S1B) of HBEC1, -2, and -26 cell lines were comparable. In contrast, the effect of BRG1-KO in inducing newly acquired anchorage-independent growth capability in soft agar showed clear heterogeneity between the three HBECs (Figure 2E and Figure S1C). While all control HBECs (HBEC1, -2, and -3), as well as the BRG1-KO HBEC1 and HBEC2 cells, formed no colonies in soft agar, BRG1-KO HBEC26 cells were transformed into anoikis-resistant cells capable of forming 97 ± 1.9 (mean ± SEM) colonies in soft agar (detailed below).

### 3.3. BRG1-LOF Enhances CS-Induced Pre-Malignant Changes

Our previous studies demonstrated that the sensitivity of HBECs to CS-induced transformation and global effects on the genome differed across cell lines [32]. Thus, the potential similarities, differences, and/or additive effects of BRG1-KO and CS exposure in lung cancer initiation were investigated using our established in vitro transformation model [32]. Overall, the results revealed that HBEC1 was the most resistant and HBEC26 the most sensitive of the three HBECs to BRG1-KO and/or CS-induced transformation. Specifically, HBEC1 formed no colony in soft agar after BRG1-KO or partial (6-week) or complete (12-week) CS exposure, or when BRG1-KO was followed by 6 weeks (wks) of CS exposure (Figure 2E). BRG1-KO HBEC1 cells subjected to the complete (12-week) CS exposure were also barely transformed (they formed no or less than three very small colonies in soft agar). HBEC2 also formed no soft agar colony after BRG1-KO or 6 wks of CS exposure alone, but 12 wks CS exposure alone resulted in a moderate number of colonies (42.3 ± 1.3, mean ± SEM). In contrast, BRG1-KO HBEC2 cells exposed to 6 wks or 12 wks of CS on average produced 85.0 ± 9.7 and 181.3 ± 5.8 colonies, respectively, indicating a synergistic (more than additive) effect of BRG1-KO in sensitizing HBEC2 cells to a quicker and stronger CS-induced transformation compared to the BRG1-WT (Figure 2E and Figure S1C). HBEC26 was the most sensitive of the three HBECs to BRG1-KO and/or CS-induced transformation, as BRG1-KO and 6 wks of CS or 12 wks of CS exposure individually and BRG1-KO followed by 6 wks or 12 wks of CS exposure respectively produced 97 ± 1.9, 8 ± 0.9, 56 ± 1.7, 119 ± 2.2, and 132 ± 6.9 colonies in soft agar (Figure 2E and Figure S1C). Taken together, these results indicate that the combination of BRG1-LOF and smoking can reduce the time to transformation with a sensitivity that clearly varies between HBEC lines from different individuals.

### 3.4. BRG1-KO Induces Transcriptome-Wide Changes

**Figure 3:**
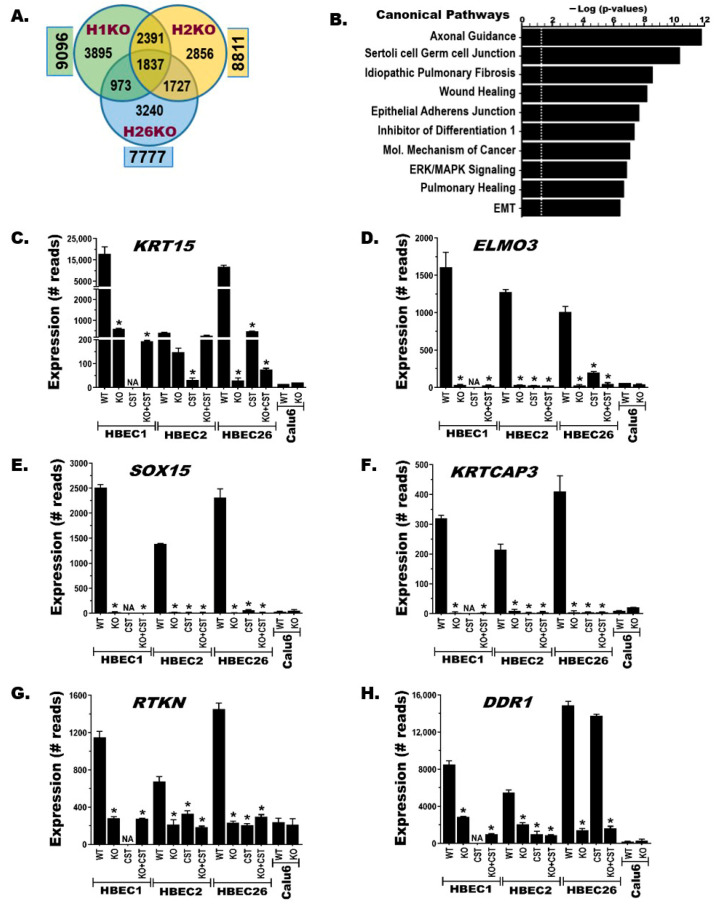
**BRG1 loss leads to transcriptome-wide expression changes.** (**A**) Venn diagram of RNA-seq-identified BRG1-KO-induced differentially expressed genes (DEGs) showing the number of common and/or unique expression changes. (**B**) Pathway analysis using the common BRG1-KO-induced DEGs showed that the top most significantly affected signaling pathways are involved in lung development, function, repair, transformation, and cancer. The white dotted line marks the threshold of significant changes. (**C**–**H**) The expression of specific genes that show common BRG1-KO-induced changes across the three HBECs revealed that the BRG1-KO-induced changes resemble the changes observed following transformation of HBECs with chronic (12-week) CS aerosol exposure (CST) and that are already found in many NSCLC tumors and cell lines such as Calu6. * *p* < 0.05. CS-exposure alone did not transform HBEC1, thus HBEC1 CST line is not available (NA).

Our previous studies established that CS or tobacco carcinogens transform HBECs through major transcriptional and epigenomic reprograming [32,50]. The genome-wide gene expression was compared between BRG1-KO and BRG1-WT HBECs, CS-transformed HBECs (CSTs), and malignant (NSCLC) cell lines using RNA-sequencing. The results showed that BRG1-KO in HBECs induced significant (false discovery rate, FDR < 0.05) expression changes in 8561 ± 567 genes (mean ± SD) compared to the corresponding BRG1-WT controls (Figure 3A). These differentially expressed genes (DEGs) generally exhibited comparable levels of increased or decreased gene expression (Figure S2A and Table S2). A Venn diagram depicting the common and distinct BRG1-KO-induced DEGs between the three HBECs revealed that 1837 (~21%) were common (Figure 3A), while 2856 to 3895 (~33–45%) DEGs were distinct for each HBEC, potentially contributing to the heterogeneity BRG1-KO HBECs showed in transformation efficiency (Figure 2E). Similarly, RNA-seq analysis of HBECs compared to CST HBECs or BRG1-KO followed by CS-exposed (KO + CST, Kcst for short) HBECs also uncovered comparably large numbers of DEGs (means ± SDs: 7791 ± 3123 and 8001 ± 868, respectively) (Figure S2B). Venn diagrams depicting the commonality and uniqueness of the CST- or Kcst-induced DEGs between HBECs (Figure S2B) and the KO-, CST-, and Kcst-induced DEGs within each HBEC cell line (Figure S2C), as well as a heatmap depicting a subset of the expression changes between HBEC1 and -2 and two BRG1-KO clones from HBEC1 (Figure S2D), demonstrate the broad changes. Pathway analysis of common BRG1-KO-induced DEGs performed using Ingenuity pathway analysis software revealed that lung development, function, damage repair (healing, fibrosis, and cell–cell junctions), transformation, and cancer-related (axonal guidance, ERK/MAPK, and epithelial-to-mesenchymal transition) signaling pathways were most significantly affected (Figure 3B). The RNA-seq data for a subset of common DEGs within the top pathways were validated using independent quantitative gene-specific qPCR (TaqMan) assays (Figure 3C–H).

### 3.5. BRG1-KO Leads to Epigenome-Wide DNA Methylation Changes

**Figure 4:**
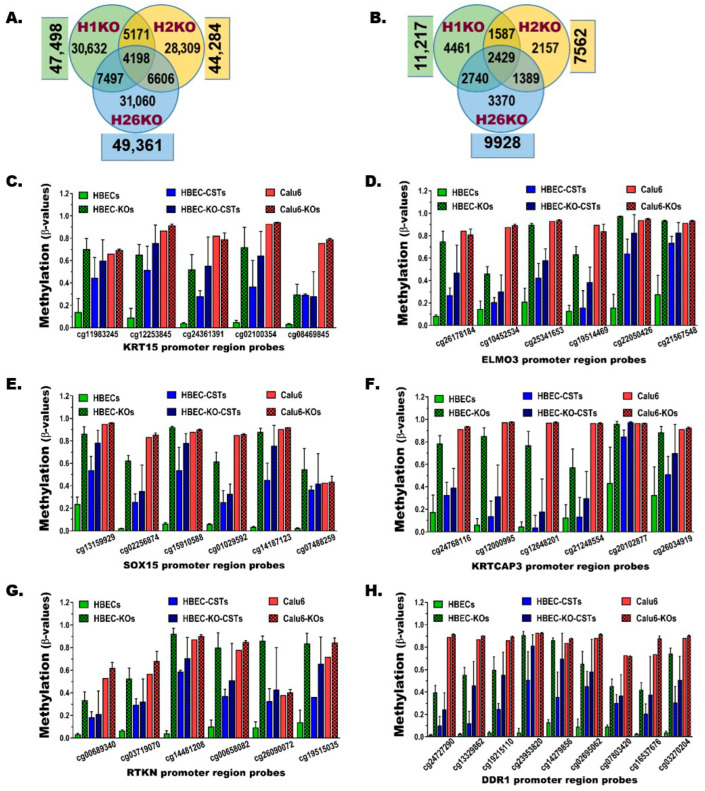
**BRG1-LOF results in broad promoter CpG methylation changes.** Evaluation of the EPICv2.0 data for genome-wide DNA methylation changes across gene promoter regions uncovered a large number of differentially methylated probes (DMPs) with the methylation level changed by ≥20%. (**A,B**) The Venn diagrams show (**A**) the number of DMPs and (**B**) the number of hypermethylated genes (HMGs) whose promoters contain multiple DMPs with increased methylation levels in the three HBECs. (**C**–**H**) The figures depict the levels of DNA methylation changes across the promoter regions of some HMGs whose expressions were also commonly repressed or silenced in the various HBEC lines (see Figure 3C–H). The data for multiple probes within the promoter region of each gene (X-axis) and the levels of methylation β-values (Y-axis) are shown. Similar to the expression changes, the methylation of these genes also shows that the BRG1-KO-induced changes resemble those found in the CST HBECs and those already present in NSCLC (e.g., Calu6).

The impact of BRG1-KO on cytosine preceding guanine (CpG) DNA methylation was investigated using the Infinium MethylationEPICv2.0 BeadChip (EPICv2.0). Although EPICv2.0 interrogates the methylation of ~930,000 sites (probes) across the genome, our analysis for this study focused on ~390,000 probes located from the 1500 bp upstream of transcription start sites (TSS1500) to the 3′-end of the first exons, where most gene promoters reside. This is because CpG methylation in promoter regions blocks the binding of transcription factors, leading to repression or complete silencing of gene expression [39,51]. Using ≥20% changes in methylation as a threshold to define a differentially methylated probe (DMP), BRG1-KO in HBEC1, -2, and -26 cell lines led to 47,048 ± 2097 DMPs (mean ± SD), including 4198 DMPs common to all three HBECs (Figure 4A, Table S3). These DMPs (increased or decreased) are distributed across the genome (promoter regions of 24,236 ± 681 genes, mean ± SD), including 8436 genes in common between the three HBECs (Figure S3A). To focus on genes that are significantly repressed or completely silenced by CpG methylation, a hypermethylated gene (HMG) was defined in this study as a gene with ≥3 or ≥20% of all interrogated promoter probes showing ≥20% increased methylation. Based on this stringent definition, BRG1-KO in the three HBECs resulted in hypermethylation of 9569 ± 1854 genes, including 2429 that were common to all three (Figure 4B, Table S3). The increased methylation of multiple consecutive probes across the promoter regions of some of these HMGs is shown in Figure 4C–H. The complete silencing or significant repression of these HMGs following BRG1-KO (Figure 3C–H) clearly show the inverse relationship between promoter hypermethylation and gene expression. Similarly, dense CpG hypermethylation changes were found in BRG1-KO HBECs exposed to CS (Kcst) as well as BRG1-KO in NSCLC cell lines (Calu6 and H358) (Table S3, Figure S3B–D). The fact that BRG1-KO-induced hypermethylation changes in the HBECs were more extensive compared to the NSCLC cell lines likely stems from the fact that many of these changes were already established in the cancer cells prior to BRG1-KO (Calu6 in Figure 4C–H).

### 3.6. BRG1-KO Promotes Epithelial-to-Mesenchymal Transition (EMT)

**Figure 5:**
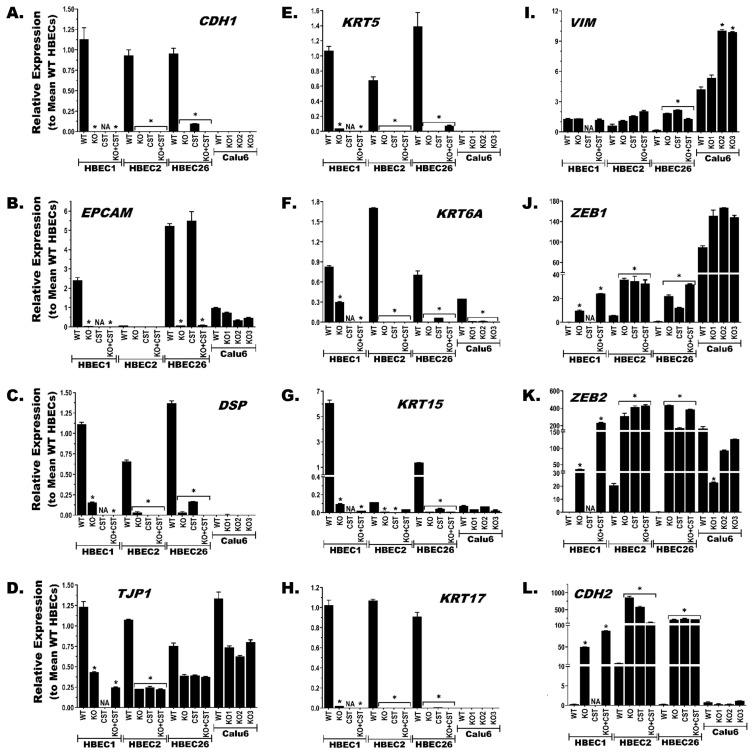
**BRG1-LOF induces epithelial-to-mesenchymal transition (EMT)**. RNA-seq-uncovered changes in the expression of major regulators/markers of EMT genes (Figure S4) were confirmed using quantitative gene-specific expression (Taqman) assays. Downregulation of well-established epithelial marker (**A**–**D**) and many keratin (**E**–**H**) genes in the BRG1-KO compared to the BRG1-WT isogeneic control HBECs revealed that these cells lose their epithelial phenotype following BRG1-LOF. Conversely, the increased expression of many EMT inducer and mesenchymal marker genes (**I**–**L**) in the BRG1-KO cells compared to the WT controls indicates that BRG1-LOF leads HBECs to gain the mesenchymal phenotype. Similar to the other changes (see Figure 3 and Figure 4), these BRG1-KO-induced EMT changes clearly transform the HBECs towards the expression profiles found in CST and NSCLC cells. * *p* < 0.05. CS-exposure alone did not transform HBEC1, thus HBEC1 CST line is not available (NA).

The role of EMT in CS-induced transformation of HBECs has been demonstrated in our published studies [32,50]. The cell morphology changes following BRG1-KO (Figure 2C) and pathway analysis of the most significant gene expression changes (Figure 3B) indicate that EMT may also play a role in the BRG1-KO-mediated transformation of HBECs. Evaluation of the RNA-seq data for changes in the major regulators and markers of EMT genes supported this proposition (Figure S4), and the changes were confirmed using quantitative gene-specific expression assays (Figure 5). Expression of well-established epithelial marker genes such as E-cadherin (CDH1), epithelial cell adhesion molecule (EPCAM), desmosome (DSP), and tight junction protein-1 (TJP1) were all downregulated in the BRG1-KO compared to the isogeneic control cell lines (Figure 5A–D and Figure S4A–D). Similarly, many keratin genes that are expressed in lung epithelial cells were significantly reduced in the BRG1-KO compared to the control lines, indicating that these cells lose their epithelial phenotype following BRG1-LOF (Figure 5E–H and Figure S4E–H). Conversely, the expression of many EMT inducer and mesenchymal marker genes was upregulated in the BRG1-KO compared to the BRG1-WT isogenic lines, indicating that BRG1-LOF leads to gain of the mesenchymal phenotype (Figure 5I–L and Figure S4L). These mesenchymal marker genes include Vimentin (VIM), zinc finger E-box-binding homeobox 1 and 2 (ZEB1 and ZEB2), N-cadherin (CDH2), Twist family transcription factor 1 and 2 (TWIST1 and TWIST2), and Snail family transcriptional repressor 1 (SNAI1). Similar to our earlier observations on the pattern and magnitude of other BRG1-KO-, CST-, and Kcst-induced alterations, these EMT-related changes also start to mirror the expression profile found in NSCLC cells (e.g., Calu6; Figure 5 and Figure S4).

### 3.7. BRG1-LOF Creates Therapeutic Vulnerabilities to Epigenetic Drugs

**Figure 6:**
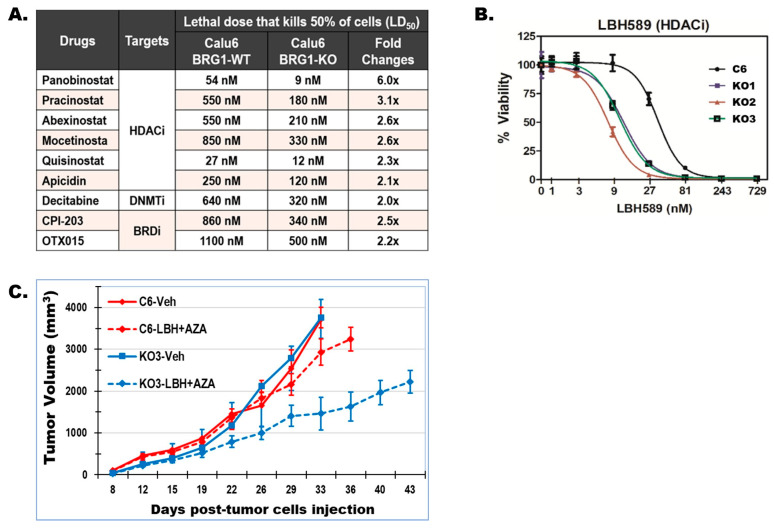
**BRG1-KO sensitizes NSCLC cells to epigenetic drugs.** (**A**) The efficacy of 152 epigenetic drugs was screened against BRG1-KO vs. WT isogenic control Calu6 (NSCLC) cell lines in vitro. The results show that BRG1-KO cells have 2-fold or higher increased sensitivity to various histone deacetylase (HDAC), bromodomain (BRD), and DNA methyltransferase (DNMT) inhibitors. (**B**) Of these, the pan-HDAC inhibitor Panobinostat (LBH589) showed the strongest (on average, 6-fold increased) efficacy against three different BRG1-KO clones (KO1, KO2, and KO3) compared to the BRG1 protein-expressing Calu6 control (C6). (**C**) As HDAC inhibitors are most effective when combined with DNMT inhibitors (see text), the in vivo efficacy of LBH589 was investigated in combination with the DNMT inhibitor 5-Azacytidine (5-AZA). Subcutaneous xenografts derived from BRG1-WT (C6) and BRG1-KO (KO3) Calu6 lines were generated and tumor-bearing mice were treated as described. While the BRG1-KO tumors treated with the LBH589 and 5-Aza combination (LBH + 5AZA) showed the slowest tumor growth and the animals showed longer survival, the differences were not statistically significant and tumors in all groups grew throughout the treatment period.

The potential impact of BRG1-KO and the resulting broad gene expression and epigenetic changes in sensitizing lung cancer cells to epigenetic therapy were screened using a library of 152 epigenetic drugs against BRG1-WT and KO Calu6 lines. The results revealed the increased sensitivity of BRG1-KO cells to various histone deacetylase (HDAC), bromodomain (BRD), and DNA methyltransferase (DNMT) inhibitors (Figure 6A). Among these, the universal HDAC inhibitor Panobinostat (LBH589) showed a 6-fold increased potency against BRG1-KO compared to BRG1-WT Calu6 cell lines. The lethal dose of LBH589 that kills 50% of the cells (LD50) was reduced from 54 nM against the BRG1-WT Calu6 cell line (C6) to an average of 9 nM (7–11 nM) against three different Calu6 BRG1-KO clones (KO1, KO2, and KO3; Figure 6B). Whether the increased in vitro sensitivity of BRG1-KO cells leads to meaningful in vivo efficacy was investigated using subcutaneous xenografts derived from BRG1-KO (KO3) versus BRG1-WT control (C6) Calu6 lines in nude mice. Based on our previous studies showing that HDAC and DNMT inhibitors produce better efficacy against lung cancer when used together [52], a combination of LBH589 and 5-Azacytidine (5-Aza) was used (Table S4). After one week of tumor growth, the mice bearing each tumor type (C6 or KO3) were treated 3 times/week for six consecutive weeks with intraperitoneal (i.p) injection of 0.5% (*v*/*v*) DMSO in DPBS (vehicle) or a combination of 4 mg/kg LBH589 and 2 mg/kg 5-Aza (LBH + AZA). Tumor growth and body weight were measured twice/week, and the mice were sacrificed at the end of the study, one week after the last (sixth) treatment, or earlier when the tumor volume exceeded 2000 mm^3^. The treatments were well-tolerated, and the mice bearing BRG1-KO (KO3)-derived tumors and treated with LBH + AZA showed the slowest tumor growth and longer survival compared to the other groups (Figure 6C). However, despite the increased sensitivity of BRG1-KO calu6 to both LBH589 and 5-AZA in vitro (Figure 6A,B) and the relatively smaller sizes of the BRG1-KO tumors in the LBH + AZA-treated mice (Figure S4), the effects were not significantly different and none of the treatments was able to arrest the highly aggressive growth of Calu6-derived tumors.

## 4. Discussion

These studies show that BRG1 loss is frequent in NSCLC and drives the transformation of lung epithelial cells, leading them to acquire properties of pre-malignant cells, indicating a potential role in lung cancer initiation. BRG1 knockout (KO) consistently transformed normal human lung epithelial cells (HBECs) to stable ‘cancer-cell-like’ morphology, gene expression, and DNA methylation profiles displaying epithelial-to-mesenchymal changes similar to those found in NSCLC cells. In addition to these common changes, BRG1-KO also led to anoikis-resistant (anchorage-independent) growth capability that varied between HBECs derived from different humans. Interestingly, both the common and distinct BRG1-KO-induced changes largely resemble the chronic cigarette smoke (CS)-induced transformation of HBECs we previously reported and reproduced in this study [32,50]. The genetic (BRG1-KO and -LOF) and environmental carcinogen (CS)-induced changes in HBECs share some common pathways and when combined produce additive to synergistic effects, transforming different HBECs. The impact of BRG1-LOF and its downstream changes on the creation of therapeutically targetable vulnerabilities in NSCLC was also investigated using a library of epigenetic drugs. BRG1-KO in Calu6 (a BRG1-WT NSCLC cell line) significantly increased its sensitivity to multiple drugs, including inhibitors of histone deacetylases (HDACs) and DNA methyltransferases (DNMTs), in vitro. However, the in vivo efficacy of the most promising combinations of the pan-HADC and DNMT inhibitors (LBH589 and 5-Aza, respectively) only led to marginally better suppression of subcutaneous tumors derived from the BRG1-KO compared to BRG1-WT Calu6. Taken together, these studies for the first time demonstrate the role of BRG1-LOF in the transformation and potentially early initiation of human lung epithelium-derived cancer via major epigenomic and transcriptomic reprogramming. Although BRG1-KO increased the sensitivity of NSCLC cells to epigenetic drugs, the effect was not strong enough to reverse or arrest xenograft tumor growth. Combining epigenetic drugs with previously identified therapeutic vulnerabilities of BRG1-LOF cancers (such as SMARCA2-degraders or inhibitors of Aurora kinase, CDK4/6, oxidative phosphorylation, or others) [23,25,26,27,28,30] will likely yield greater responses and warrants further investigation.

BRG1 mutation and loss of function are important diagnostic abnormalities for the unique ovarian (SCCOHT) and thoracic (SMARCA4-DTS) cancers and are detected in increasing numbers of other malignancies [5,6,7]. In NSCLC, BRG1 mutation is found in ~10% of cases, with comparable frequencies in tumors from smokers and never-smokers [4,8,13,14]. Our protein analysis showed that BRG1 function was compromised (no or barely detectable expression) in nearly half (~47%) of the 30 NSCLC cell lines evaluated. Similarly, a recent analysis of 39 commonly used lung adenocarcinoma cell lines revealed BRG1 protein loss in 32% of cases, while 42% showed coding region or splice site mutation [49]. These findings indicate that the true prevalence and impact of BRG1-LOF in NSCLC could be higher than current estimates based on coding region mutations that ignore BRG1 loss due to mutations outside of the coding region and/or non-genetic (e.g., epigenetic) abnormalities. In addition, protein analysis of lung cancer cell lines captures the critical cancer cell-specific BRG1 loss that could be masked in tumors by expression from stromal and/or tumor-infiltrating normal cells. Thus, combining BRG1 protein levels detected specifically within the tumor cells along with mutation analysis to detect coding region mutations that may result in LOF but not loss of BRG1 protein is needed to capture the complete picture. Fortunately, reliable BRG1 mutation and immunohistochemistry assays are available and currently used in the clinic to support the diagnosis of SCCOHT, SMARCA4-DTS, and some MRTs and should be extended for evaluating BRG1 status in NSCLCs [53].

Our previous studies demonstrated a similar stable transformation of HBECs following chronic CS exposure in vitro [32,50]. The strong similarity between the CS- and BRG1-KO-induced transformation, including the heterogeneity of these HBECs in terms of acquiring anoikis-resistant growth capabilities, suggests that CS exposure may negatively affect BRG1 function or share some common pathways. However, the fact that BRG1 mRNA and protein expression in the CS-transformed HBECs was not significantly different from that in the control HBECs suggests that BRG1 may not be directly affected by CS exposure. A total of 29 genes, including BRG1 and its paralog BRM, encode the various proteins assembled in 10–15 different combinations of subunits to form the mammalian SWI/SNF complex [54]. As BRG1 loss alters the normal composition and functions of SWI/SNF, CS exposure may produce similar downstream effects by affecting one or more of these other subunits. While BRG1 and BRM form the single ATPase subunit of all SWI/SNF complexes in a mutually exclusive manner, different combinations of the other subunits support distinct functions. Thus, CS-induced changes to any of the SWI/SNF subunits could lead to BRG1-LOF-like changes without directly affecting BRG1 functions. As SWI/SNF mutation is one of the most common abnormalities in cancer (found in >20% of all cancers) [9,10,12], the plausible hypothesis that it might be a common target of CS-induced damage needs to be investigated.

The role of BRG1-LOF in lung cancer development was recently shown using the Kras and p53 mutant (KP) genetically engineered mouse model (GEMM) [55]. Since Kras and p53 mutations are critical drivers of lung cancer in this model, the role of BRG1-LOF in lung tumorigenesis without these two or other known cancer-driver mutations was not evaluated. Our data for the first time clearly demonstrated that BRG1-LOF in human lung epithelial cells induced stable pre-malignant transformation. These BRG1-KO-induced changes included a morphological shift from epithelial to elongated ‘fibroblast-like’ cells with anoikis-resistant growth capabilities and major epigenome- and transcriptome-wide reprograming that activated EMT and other cancer-related pathways. Comparisons of selected gene expression and DNA methylation changes between BRG1-WT versus KO HBECs and NSCLC cell lines indicate that the changes in the BRG1-KO HBECs mirror those commonly found in many NSCLC cell lines and primary tumors. Thus, depending on when this critical ATPase of the master chromatin remodeling SWI/SNF complex is lost and starts affecting gene expression programs, it could play a major role in the initiation of pre-malignancy and progression of NSCLC. The loss of BRG1 in various human cancers, including nearly all SMARCA4-DTS and SCCOHT cancers and at least 10% of NSCLC cancers, which often have a typical, poorly differentiated rhabdoid morphology and highly aggressive clinical features [4,5,6,7,8], supports this premise.

## 5. Conclusions

Our results demonstrated that BRG1-LOF is frequent in NSCLC and drives the early transformation of lung epithelial cells, leading them to acquire properties of pre-malignant cells, indicating a potential role in lung cancer initiation. In addition, BRG1 loss, together with its broad downstream epigenetic and gene expression changes, sensitizes lung cancer cells to various drugs, and these therapeutic vulnerabilities must be carefully exploited to improve the treatment of this leading cause of cancer-related death worldwide.

## Data Availability

The genome-wide RNA-seq and DNA methylation data generated in this study have been deposited in the NCBI database (GEO accession number: GSE307857) and will be publicly accessible after publication of the data or the embargo, whichever comes first.

## Supplementary Materials

The following supporting information can be downloaded at https://www.mdpi.com/article/10.3390/cancers17183092/s1: Figure S1: Effects of BRG1 knockdown (KD) or knockout (KO) in normal lung epithelial cell lines. (A) BRG1 was transiently knocked down using BRG1-specific siRNA, and the changes in its mRNA (bar graph) and protein (Western blotting) expression are shown. (B) Effects of BRG1 loss on cell migration were compared between BRG1-WT and BRG-KO HBEC lines using a colorimetric trans-well cell migration assay as described, and pictures of the migrated cells are shown. (C) The impacts of BRG1-LOF and/or in vitro exposure to chronic cigarette smoke aerosol on anchorage-independent growth in soft agar were evaluated, and pictures of colonies for HBEC2 and HBEC26 are shown. Figure S2: BRG1-KO-induced genome-wide gene expression. (A) Volcano plot showing RNA-seq-uncovered gene expression changes following BRG1-KO in HBEC1. (B–C) Venn diagrams showing the numbers of common and unique gene expression changes (B) between HBECs following CS-induced transformation (CST) alone or after BRG1-KO (Kcst) and (C) within the same HBEC line following BRG1-K, CST, or both (Kcst). (D) A heatmap displaying a subset of the BRG1-KO-induced gene expression changes between HBECs. Figure S3: BRG1-KO-induced genome-wide DNA methylation changes. (A–D). Venn diagrams showing the common and distinct DNA methylation changes. (A) Differentially methylated probes (DMPs) following BRG1-KO in HBECs. (B) Hypermethylated genes (HMGs) between CS-transformed HBECs without BRG1-KO (CST) or after BRG1-KO (Kcst). (C) HMGs within the same HBEC lines following BRG1-KO, CST, or BRG1-KO followed by CS exposure (Kcst). (D) HMGs with significant repression of expression in HBECs transformed by BRG1-KO alone or followed by CS exposure (Kcst). Figure S4: BRG1 loss leads to epithelial-to-mesenchymal transition (EMT). (A–L) The changes in the expression of major regulators and markers of EMT genes identified from the RNA-seq data. Downregulation of well-established (A–D) epithelial marker genes and (E–H) many keratin genes in the BRG1-KO compared to the isogeneic control lines indicates that these cells lose their epithelial phenotype following BRG1-LOF. Conversely, the increased expression of many EMT inducer and mesenchymal marker genes (I–L) in the BRG1-KO compared to BRG1-WT isogenic control lines indicates that BRG1-LOF leads to gain of mesenchymal phenotype. Similar to the earlier observations (with other genes, see Figure 3), the changes in these EMT genes in the BRG1-KO, CST, and Kcst HBECs clearly follow similar patterns and show change towards the expression profile found in the NSCLC cell line Calu6. Figure S5: BRG1 loss sensitizes NSCLC cells to epigenetic therapy in vitro and in vivo. (A–D). Mice bearing subcutaneous xenografts derived from the BRG1-WT calu6 cell line (Calu6) and treated with (A) a vehicle or (B) a combination of LBH589 and 5-Azacytidine (LBH + 5AZA), as well as tumors from the BRG1-KO calu6 cell line (KO3) treated with (C) a vehicle or (D) LBH + 5AZA, are shown. Table S1: BRG1 mutation and protein expression of 47 human lung epithelium-derived cell lines at different stages of malignancy (normal, transformed, or cancer) [56,57]. Table S2: Genome-wide gene expression changes following BRG1-KO and/or chronic cigarette smoke aerosol exposure (CST) of HBEC cell lines. Table S3: Genome-wide DNA methylation analysis of BRG1-KO- and/or CS-induced changes across gene promoter regions of HBECs and NSCLC cells. Table S4: Experimental design for epigenetic therapy of BRG1-KO NSCLC-derived subcutaneous xenografts in nude mice. Supplementary File S1: The original without uncropped Western Blot (WB) images.

## Abbreviations

The following abbreviations are commonly used in this manuscript:

BRG1	Brahma-related gene-1, also called SMARCA4
BRM	Brahma, also called SMARCA2
BAF	BRG1/BRM-associated factors, also called SWI/SNF
CRISPR	Clustered Regularly Interspaced Short Palindromic Repeats
CS	Cigarette smoke
CST	Cigarette smoke-transformed
EMT	Epithelial-to-mesenchymal transition
HBECs	Human bronchial epithelial cell lines
LOF	Loss of function
NSCLC	Non-small cell lung cancer
SCCOHT	Small cell carcinoma of the ovary hypercalcemic type
SWI/SNF	Switch/Sucrose non-fermentable
SMARCA2	SWI/SNF-related BAF chromatin remodeling complex subunit ATPase 2
SMARCA4	SWI/SNF-related BAF chromatin remodeling complex subunit ATPase 4
SMARCA4-DTS	SMARCA4-deficient thoracic sarcoma
