# Supplementary material for: BRG1 Loss Is Frequent in Lung Cancer and Transforms Lung Epithelial Cells via Transcriptional and Epigenetic Reprograming
Source: Cancers (Basel). 2025 Sep 22;17(18):3092. doi: 10.3390/cancers17183092 (PMC12468026; doi:10.3390/cancers17183092)
Supplement: Supplementary file 1 [file cancers-17-03092-s001.zip › cancers-3797039-supplementary(final).pdf]

cancers MDPI

Supplementary Materials

Table S1. BRG1 mutation and protein expression of 47 human lung epithelia derived cell lines at different stages malignancy (normal, transformed, or cancer).

Cell line		Mutation *	Protein **	References
Normal human bronchial epithelial cell lines (HBECs). (n = 7)	HBEC1	ND	Yes	
	HBEC2	ND	Yes	
	HBEC3	WT	Yes	[1]
	HBEC4	ND	Yes	
	HBEC13	ND	Yes	
	HBEC14	ND	Yes	
	HBEC26	ND	Yes	
Normal human small airway epithelial cell lines (HSAECs). (n = 5)	HSAEC13	ND	Yes	
	HSAEC19	ND	Yes	
	HSAEC22	ND	Yes	
	HSAEC30	ND	Yes	
	HSAEC31	ND	Yes	
Cigarette smoke transformed (CST) HBEC or HSAEC cell lines. (n = 5)	HBEC2-CST	ND	Yes	
	HBEC4-CST	ND	Yes	
	HBEC14-CST	ND	Yes	
	HBEC26-CST	ND	Yes	
	HSAEC30-CST	ND	Yes	
Non-small cell lung cancer (NSCLC). (n = 30)	A427	Mut	No	[2–4]
	A549	Mut	No	[1–6]
	Calu3	WT	No	[1–3]
	Calu6	WT	Yes	[7]
	H23	Mut	No	[1–4,6]
	H358	WT	Yes	[1–4,6]
	H441	WT	Yes	[2–4,6]
	H460	WT	Yes	[2,5]
	H520	WT	Yes	[2,5,6]
	H522	Mut	No	[2–6]
	H1299	Mut	No	[1,2,4–6]
	H1435	Mut	No	[3]
	H1568	Mut	No	[3,4]
	H1650	Mut	No	[3]
	H1703	Mut	No	[2,4–6]
	H1838	ND	Yes	
	H1993	ND	No	
	H1975	WT	Yes	[3,8]
	H2023	ND	No	[8]
	H2030	Mut	No	[3,4]
	H2085	ND	No	
	HCC78	WT	Yes	[2–4]
	H2170	WT	Yes	[6]
	H2228	WT	Yes	[3,6]
	H3255	WT	Yes	[1,4]
	HCC827	WT	Yes	[1–4]
	HCC4006	Mut	No	[1,3]
	PC9	WT	Yes	[3,4]
	SKMES1	WT	Yes	[2,6]

SW900	WT	Yes	[6]

* Mutation in the coding region based on cited references; ND (not yet determined).
** Protein expression based on our western blot analysis (see Figure 1A-B).

Table S2. Genome-wide gene expression changes following BRG1-KO and/or chronic cigarette smoke aerosol exposure (CST) of HBEC cell lines.

Comparisons		Differentially Expressed Genes (DEGs; FDR ≤ 0.05)		
**Cell lines**	**Treatments**	**All DEGs**	**Increased DEGs**	**Decreased DEGs**
**HBEC1**	WT vs KO	9096	4548	4548
	WT vs KOcst	7118	3470	3648
**HBEC2**	WT vs KO	8811	4431	4380
	WT vs CST	5583	2903	2680
	WT vs KOcst	8853	4482	4371
**HBEC26**	WT vs KO	7777	3731	4046
	WT vs CST	9999	4890	5109
	WT vs KOcst	8032	3851	4181
**Calu6**	WT vs KO	5988	3351	2637
**H358**	WT vs KO	6977	3482	3495

Table S3. Genome-wide DNA methylation analysis of BRG1-KO and/or CS induced changes across gene promoter regions of HBECs and NSCLC cells.

Comparisons		BRG1-KO and/or CS induced methylation changes *	
**Cell lines**	**Treatments**	**DMPs**	**HMGs**
**BRG1-WT vs BRG1-KO**	HBEC1	47,498	11,217
	HBEC2	44,284	7562
	HBEC26	49,361	9928
	Mean ± SD	47,048 ± 2568	9569 ± 1854
**BRG1-WT air vs CS**	HBEC2	44,463	3157
	HBEC26	36,316	6563
	Mean ± SD	40,390 ± 4074	4860 ± 1703
**BRG1-WT air vs BRG1-KO +CS**	HBEC1	57,006	4178
	HBEC2	48,232	7101
	HBEC26	53,285	9351
	Mean ± SD	52,841 ± 3596	6877 ± 2118
**BRG1-WT vs BRG1-KO**	Calu6-KO7	13,000	1797
	Calu6-KO23	17,436	2132
	Mean ± SD	15,218 ± 2218	1965 ± 168
**BRG1-WT vs BRG1-KO**	H358-KO1	20,419	4225
	H358-KO6	12,940	3622
	Mean ± SD	16,680 ± 3740	3924 ± 302

* Differentially methylated probes (DMPs) are probes (CpGs) with ≥ 20% changes in methylation. Hypermethylated genes (HMGs) are genes in which at least 3 or 20% of all probes within the promoter region show ≥ 20% increased methylation level.

Table S4. Experimental Design for epigenetic therapy of BRG1-KO NSCLC derived Subcutaneous Xenografts in nude mice.

Group	Cell line	Number of mice	Number of cells per injection site	Treatments *
1	Calu6-WT	5	2.5×10^6 cells	Vehicle
2	Calu6-WT	5	2.5×10^6 cells	LBH589 + 5-Aza
3	Calu6-KO3	5	2.5×10^6 cells	Vehicle
4	Calu6-KO3	5	2.5×10^6 cells	LBH589 + 5-Aza

* Intraperitoneal (i.p) injection of Vehicle [DMSO 0.5% (v/v) in PBS] or LBH589 (4 mg/kg) and 5-Aza (2.0 mg/kg) 3 days/week for 6 consecutive weeks.

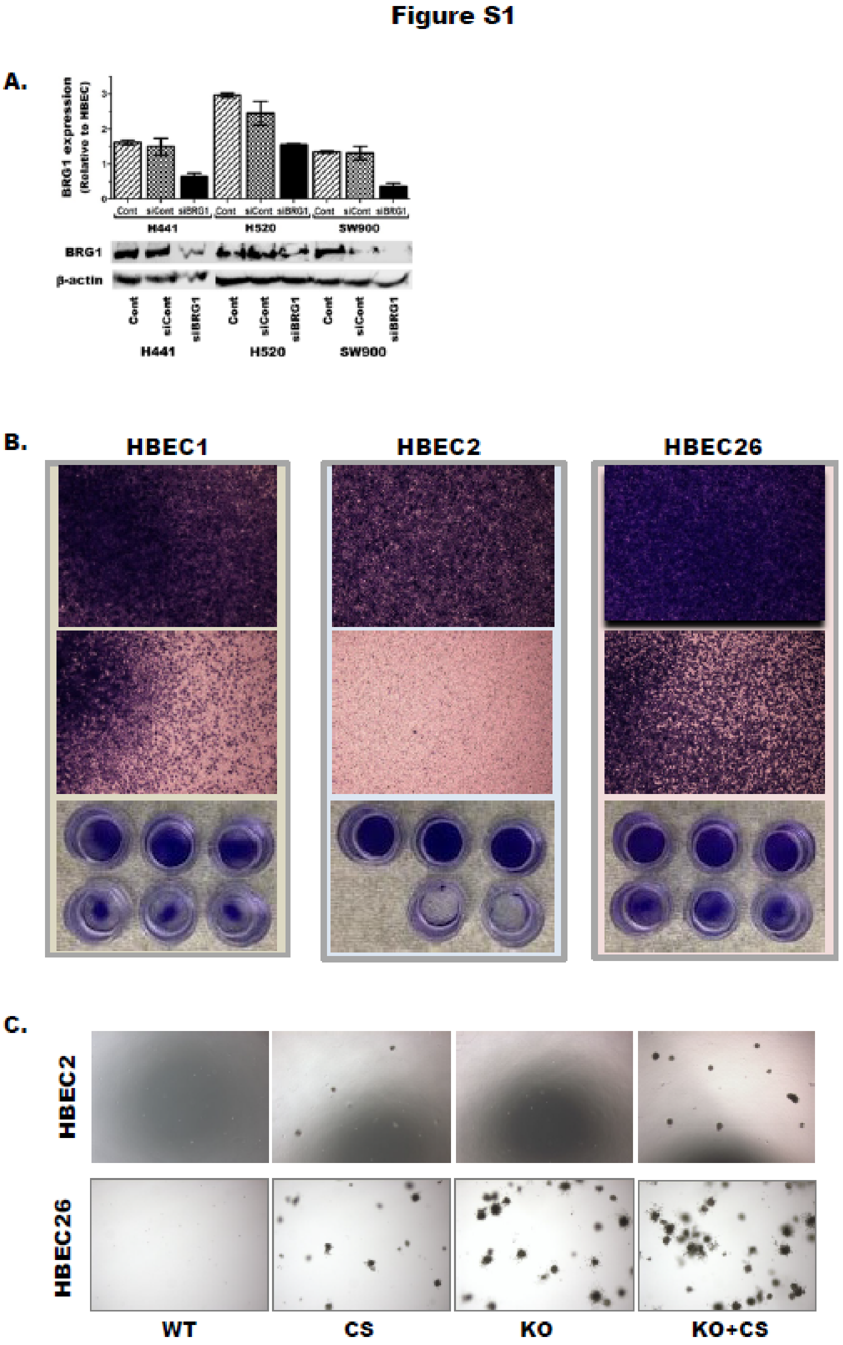

Figure S1. Effects of BRG1 knockdown (KD) or knockout (KO) in normal lung epithelial cell lines. A. BRG1 was transiently knocked down using BRG1-specific siRNA and the changes in its mRNA (bar graph) and protein (western blot) expression are shown. **B.** Effects of BRG1 loss on cell migration was compared between BRG1-WT and BRG-KO HBEC lines using a colorimetric trans-well cell migration assay as described and pictures of the migrated cells are shown. **C.** The impacts of BRG1-LOF and/or in vitro exposure to chronic cigarette smoke aerosol on anchorage-independent growth in soft agar was evaluated and pictures of colonies for HBEC2 and HBEC26 are shown.

Figure S2

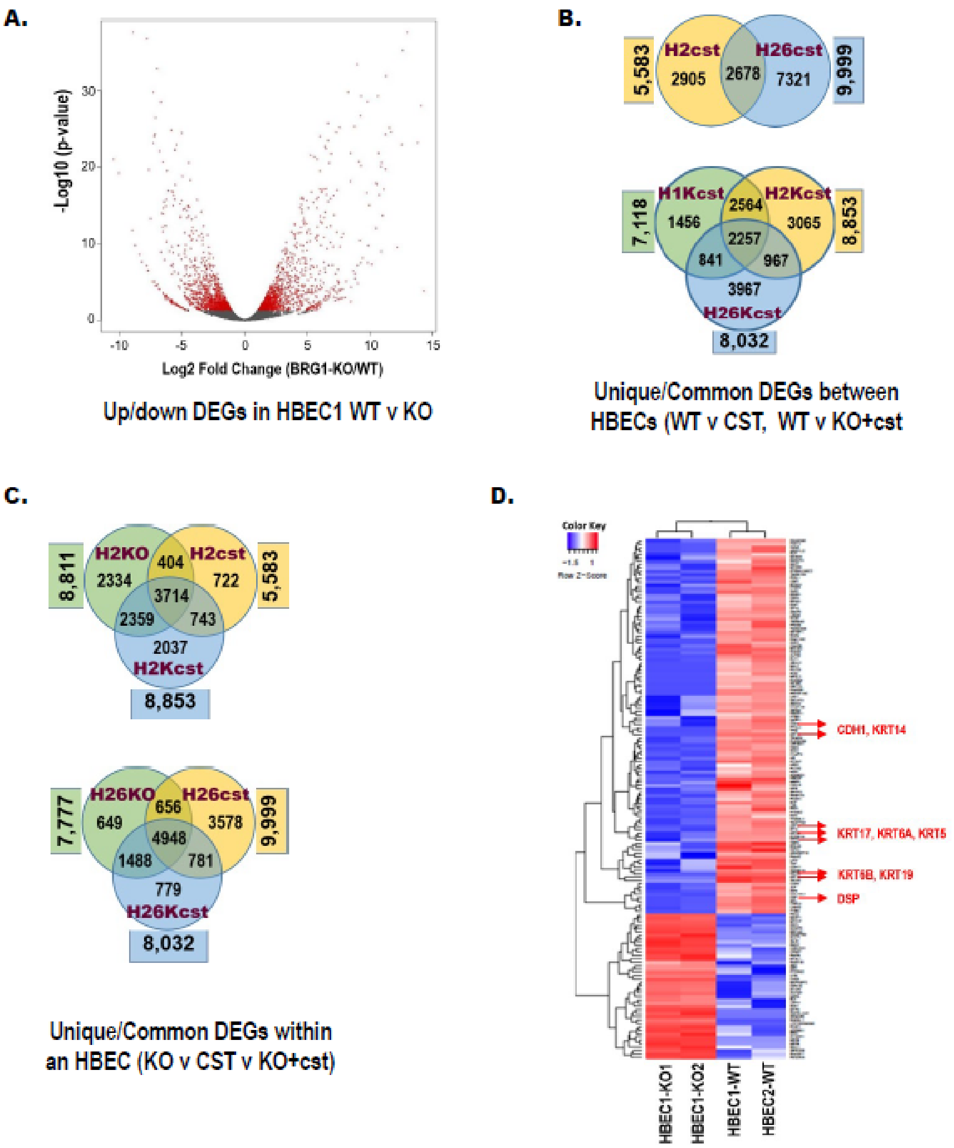

Figure S2. BRG1-KO induced genome-wide gene expression. A. Volcan plot shows RNA-seq uncovered gene expression changes following BRG1-KO in HBEC1. **B–C.** Venn diagrams showing the number of common and unique genes expression changes **(B)** between HBECs following CS-induce transformation (CST) alone or after BRG1-KO (Kcst) or **(C)** within the same HBEC line following BRG1-K), CST, or both (Kcst). **D.** A heatmap displaying a subset of the BRG1-KO induced gene expression changes between HBECs.

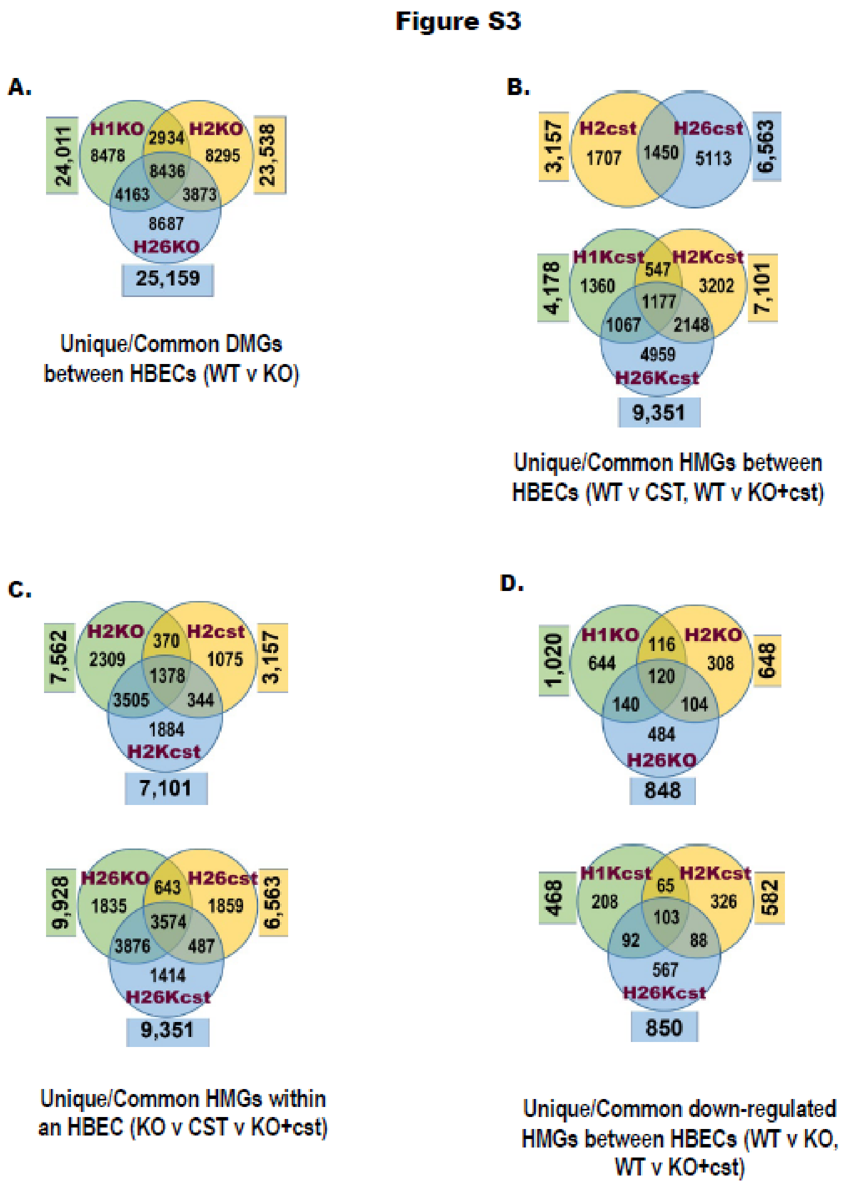

Figure S3. BRG1-KO induced genome-wide DNA methylation changes. A–D. Venn diagrams show the common and distinct DNA methylation changes. **(A)** Differentially methylated probes (DMPs) following BRG1-KO in HBECs. **(B)** Hypermethylated genes (HMGs) between CS transformed HBECs without BRG1-KO (CST) or after BRG1-KO (Kcst). **(C)** HMGs within the same HBEC lines following BRG1-KO, CST, or BRG1-KO followed by CS exposure (Kcst). **(D)** HMGs with significant repression of expression in HBECs transformed by BRG1-KO alone or followed by CS exposure (Kcst).

Figure S4

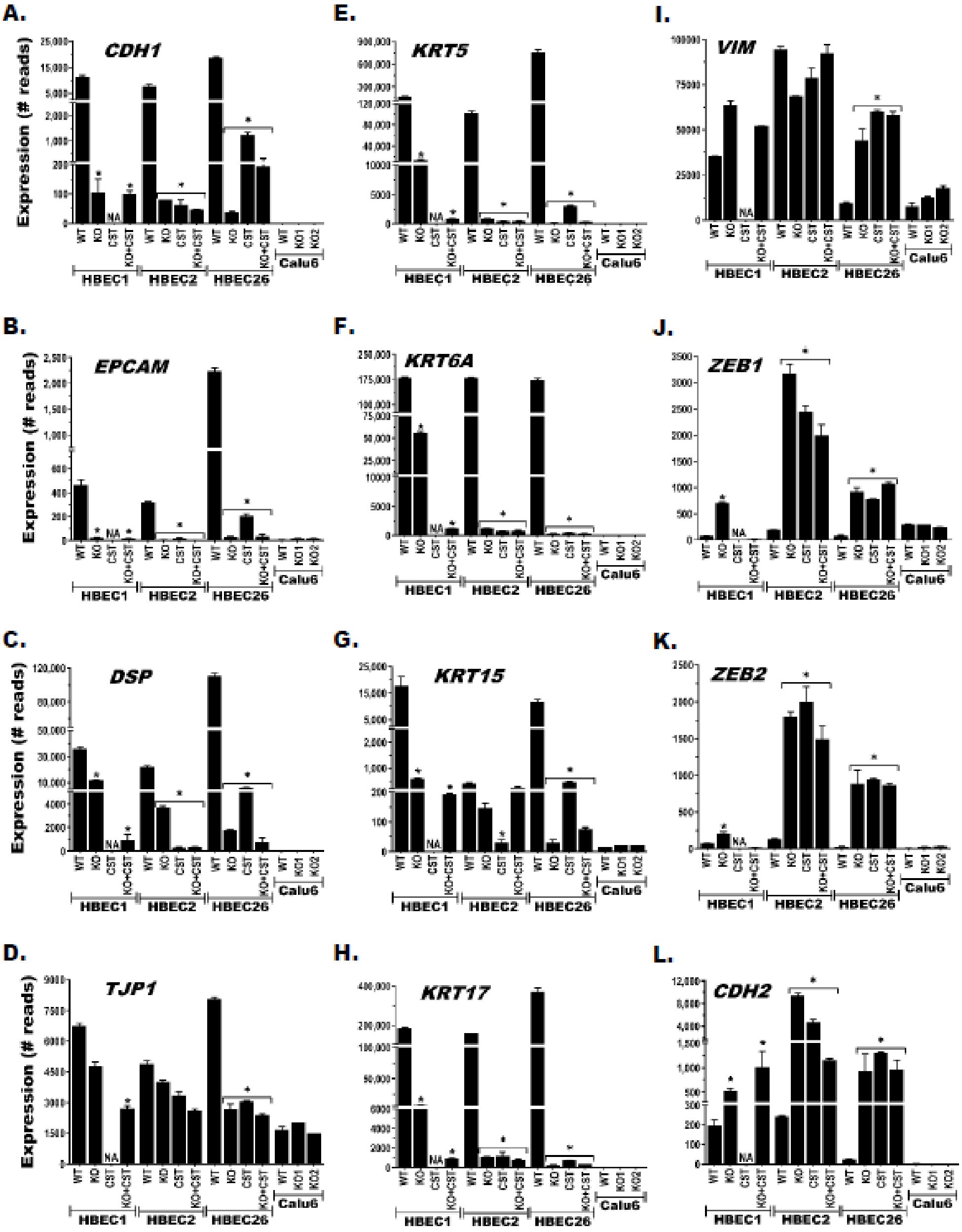

Figure S4. BRG1-loss leads to epithelial-to-mesenchymal transition (EMT). A–L. The changes in the expression of major regulators and markers of EMT genes identified from the RNA-seq data. Down regulation of well-established **(A–D)** epithelial marker genes and **(E–H)** many keratin genes in the BRG1-KO compared to the isogeneic control lines indicate that these cells are losing their epithelial phenotype following BRG1-LOF. Conversely, the increase expression of many EMT inducer and mesenchymal marker genes **(I–L)** in the BRG1-KO compared to BRG1-WT isogenic control lines indicating that BRG1-LOF leads to gain of mesenchymal phenotype. Similar to the earlier observation (with other genes, see **Figure 3**), the changes of these EMT genes in the BRG1-KO, CST, and Kcst HBECs clearly follow similar pattern and changing towards the expression profile found in the NSCLC cell line Calu6.

Figure S5

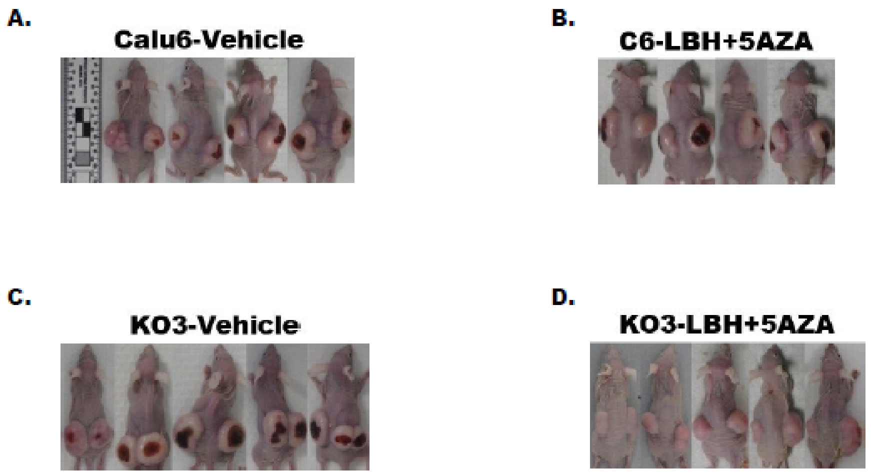

Figure S5. BRG1 loss sensitizes NSCLC cells to epigenetic therapy in vitro and in vivo. A–D. Mice bearing subcutaneous xenografts derived from BRG1-WT calu6 cell line (Calu6) and treated with **(A)** Vehicle or **(B)** a combination of LBH589 and 5-Azacytidine (LBH+5AZA) as well as tumors from BRG1-KO calu6 cell line (KO3) and treated with **(C)** Vehicle or **(D)** LBH+5AZA are shown.

References

1. Tagal, V., S. Wei, W. Zhang, et al., *SMARCA4-inactivating mutations increase sensitivity to Aurora kinase A inhibitor VX-680 in non-small cell lung cancers.* Nat Commun, 2017. **8**: p. 14098.
2. Medina, P.P., O.A. Romero, T. Kohno, et al., *Frequent BRG1/SMARCA4-inactivating mutations in human lung cancer cell lines.* Hum Mutat, 2008. **29**(5): p. 617-22.
3. Peinado, P., A. Andrades, M. Cuadros, et al., *Comprehensive Analysis of SWI/SNF Inactivation in Lung Adenocarcinoma Cell Models.* Cancers (Basel), 2020. **12**(12).
4. Xue, Y., B. Meehan, Z. Fu, et al., *SMARCA4 loss is synthetic lethal with CDK4/6 inhibition in non-small cell lung cancer.* Nat Commun, 2019. **10**(1): p. 557.
5. Oike, T., H. Ogiwara, Y. Tominaga, et al., *A synthetic lethality-based strategy to treat cancers harboring a genetic deficiency in the chromatin remodeling factor BRG1.* Cancer Res, 2013. **73**(17): p. 5508-18.
6. Orvis, T., A. Hepperla, V. Walter, et al., *BRG1/SMARCA4 inactivation promotes non-small cell lung cancer aggressiveness by altering chromatin organization.* Cancer Res, 2014. **74**(22): p. 6486-6498.
7. Berlin, M., J. Cantley, M. Bookbinder, et al., *PROTACs Targeting BRM (SMARCA2) Afford Selective In Vivo Degradation over BRG1 (SMARCA4) and Are Active in BRG1 Mutant Xenograft Tumor Models.* J Med Chem, 2024. **67**(2): p. 1262-1313.
8. Lissanu Deribe, Y., Y. Sun, C. Terranova, et al., *Mutations in the SWI/SNF complex induce a targetable dependence on oxidative phosphorylation in lung cancer.* Nat Med, 2018. **24**(7): p. 1047-1057.
