# Supplementary material for: BRG1 Loss Is Frequent in Lung Cancer and Transforms Lung Epithelial Cells via Transcriptional and Epigenetic Reprograming
Source: Cancers (Basel). 2025 Sep 22;17(18):3092. doi: 10.3390/cancers17183092 (PMC12468026; doi:10.3390/cancers17183092)
Supplement: Supplementary file 1 [file cancers-17-03092-s001.zip › Supplementary File S1.pdf]

cancers

MDPI

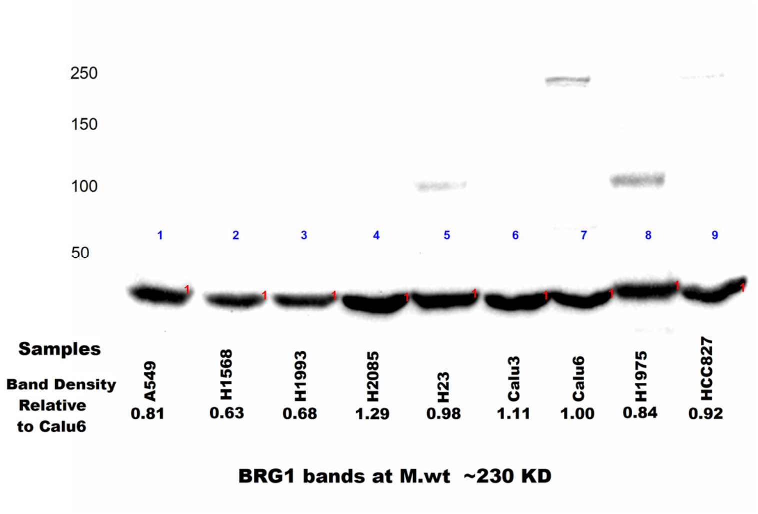

Figure 1. A B-actin(A549 to HCC827).

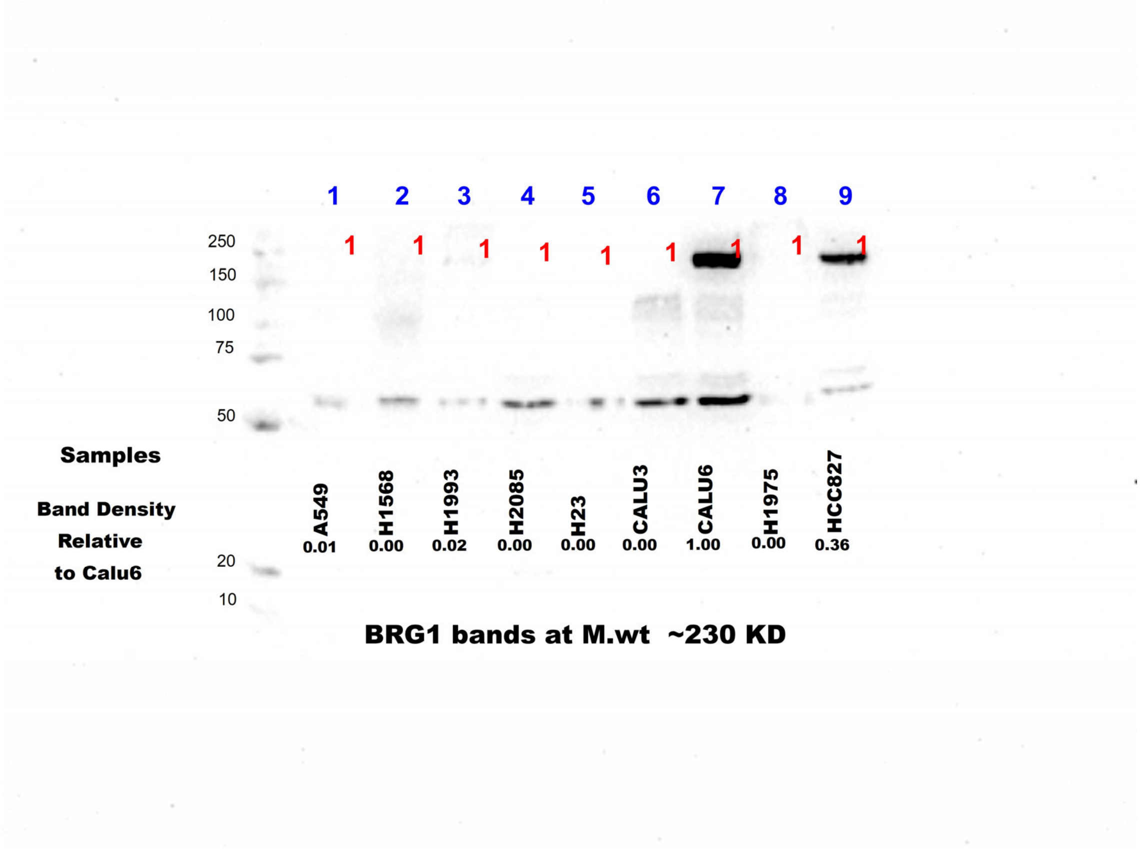

Figure 1. A BRG1(A549 to HCC827).

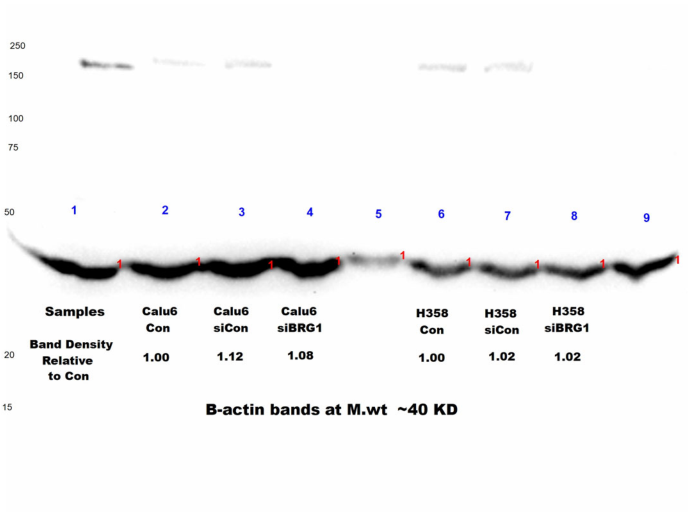

Figure 2. A B-actin(Calu6 H358).

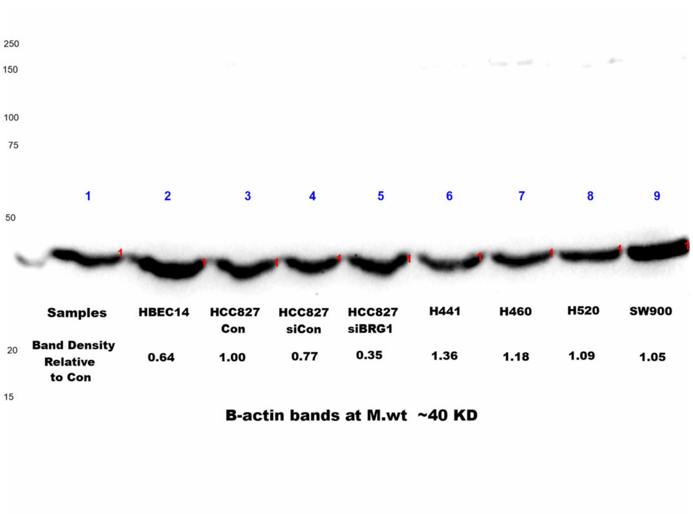

Figure 2. A B-actin(HCC827)and 4Fig1A.

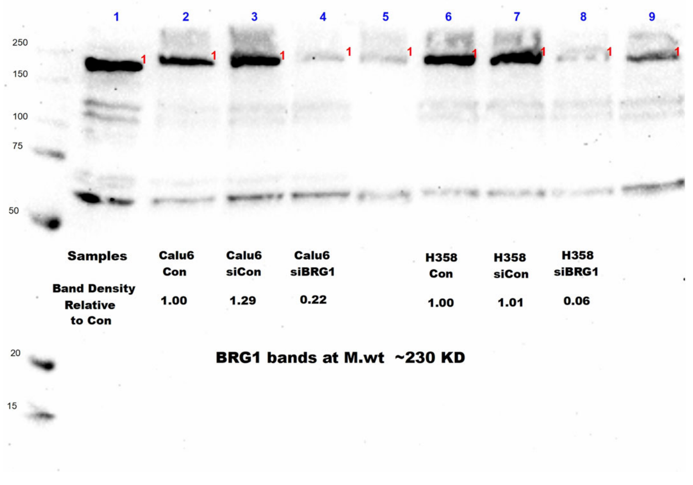

Figure 2. A BRG1(Calu6 H358).

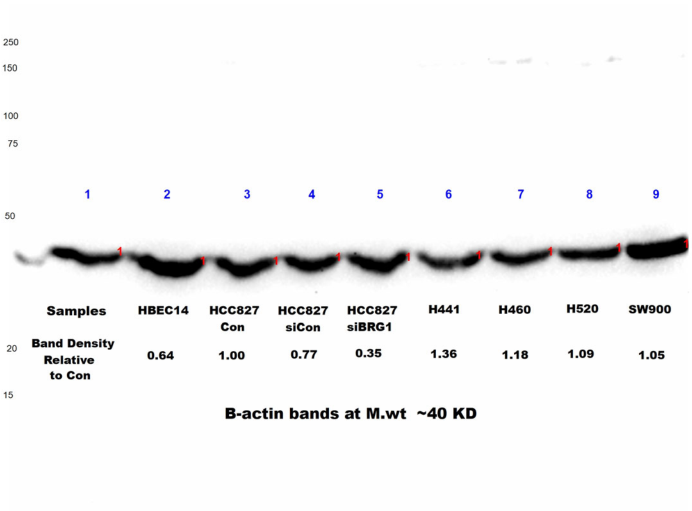

Figure 2. A BRG1(HCC827)and 4Fig1A.

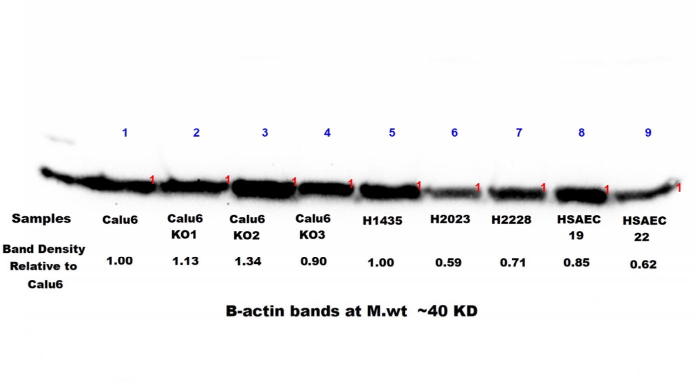

Figure 2. B B-actin(C6, KOs).

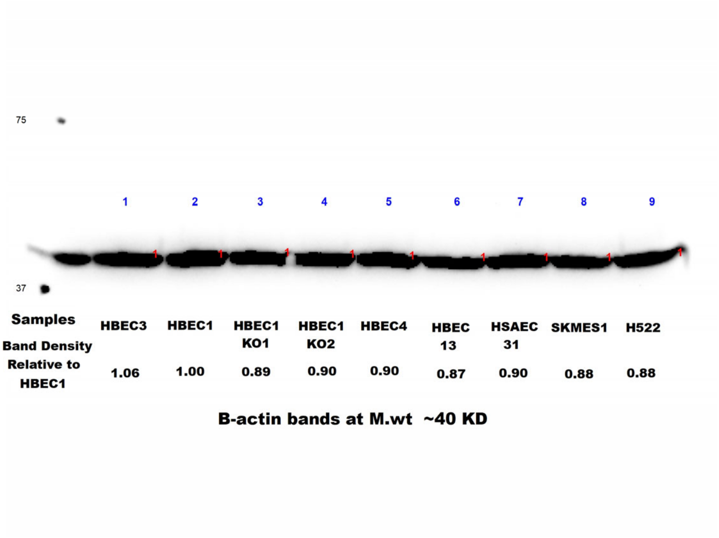

Figure 2. B B-actin(H1, KOs).

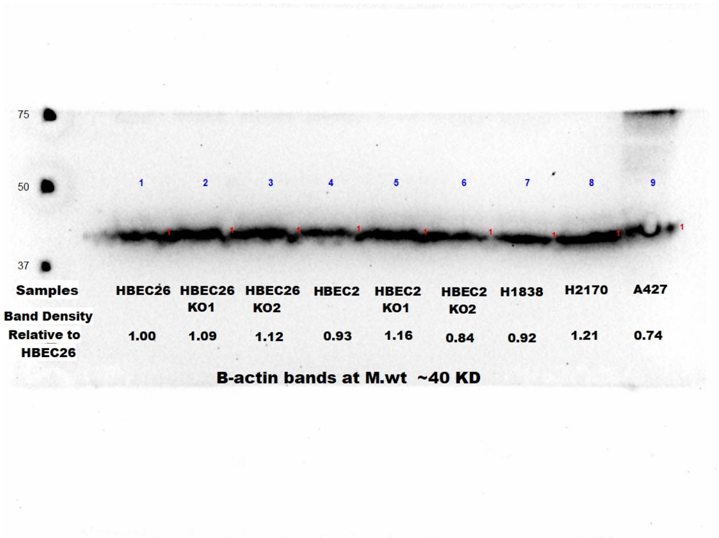

Figure 2. B B-actin(H2 KOs H26 KOs).

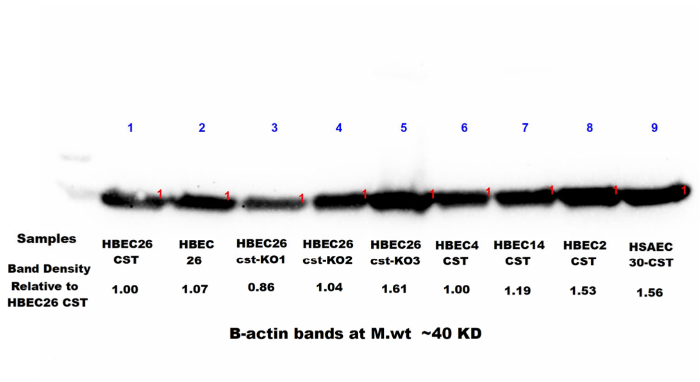

Figure 2. B B-actin(H26cst, KOs CSTs).

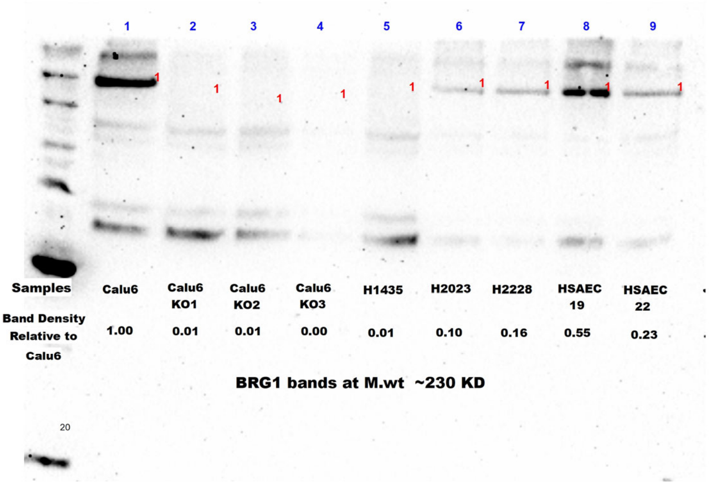

Figure 2. B BRG1 (C6, KOs).

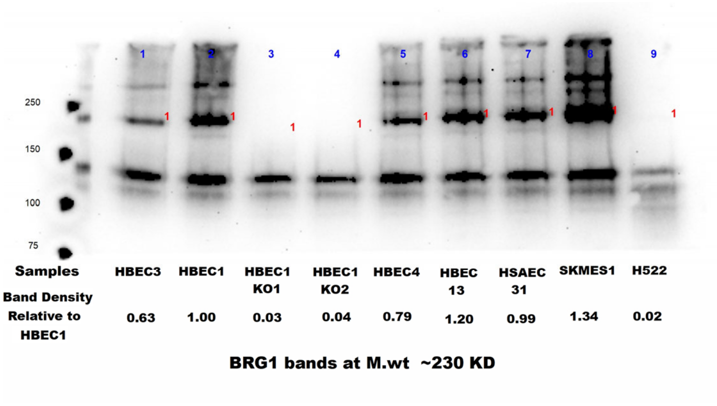

Figure 2. B BRG1(H1, KOs).

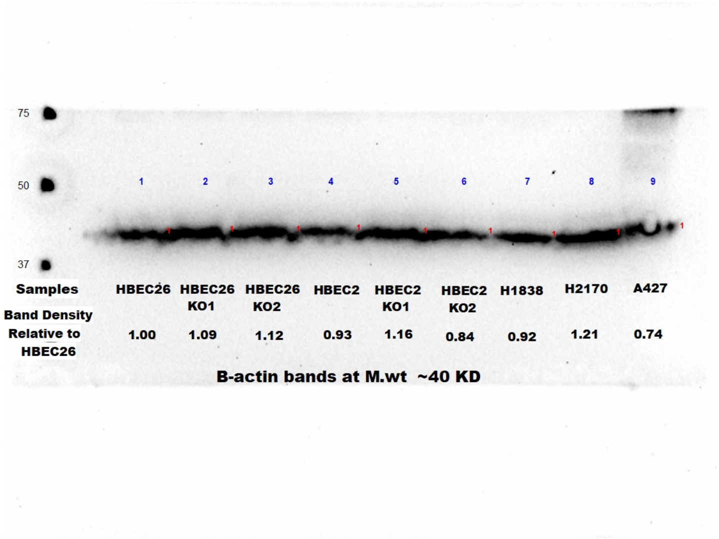

Figure 2. B B-actin(H2 KOs H26 KOs).jpg.

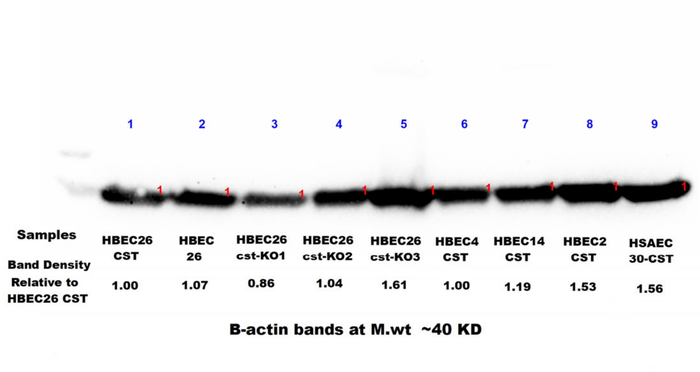

Figure 2. B B-actin(H26cst, KOs CSTs).jpg.

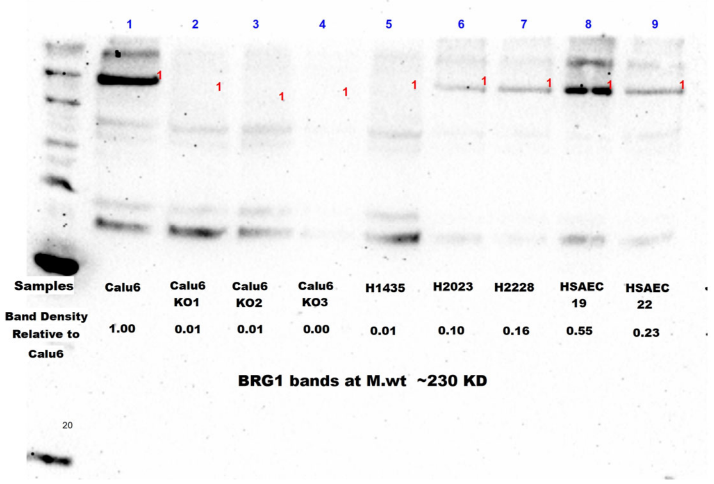

Figure 2. B BRG1 (C6, KOs).jpg.

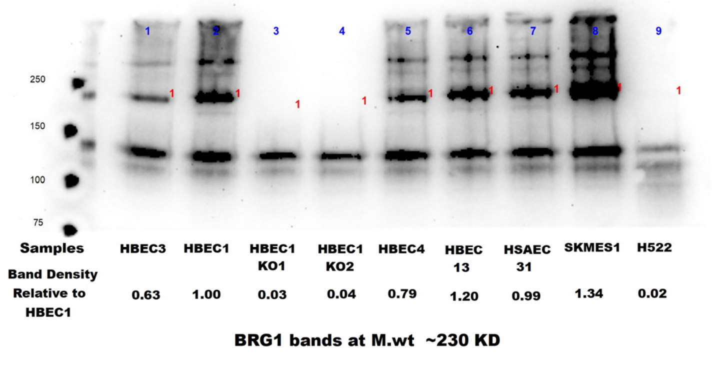

Figure 2. B BRG1(H1, KOs).

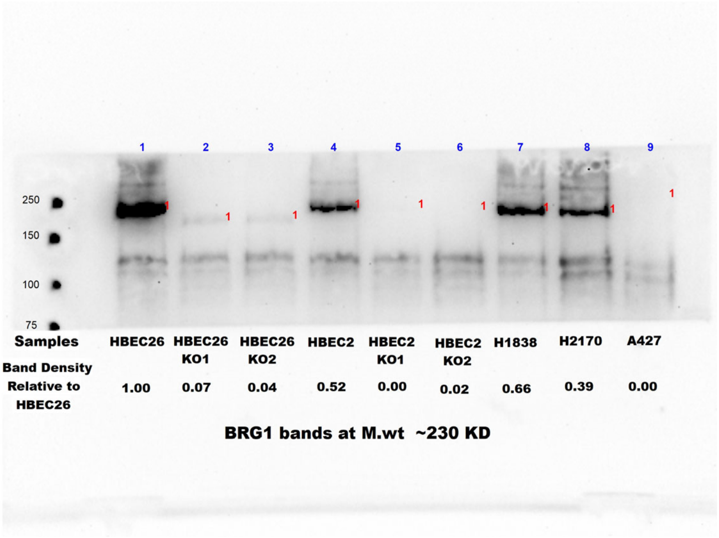

Figure 2. B BRG1(H2 KOs H26 KOs).

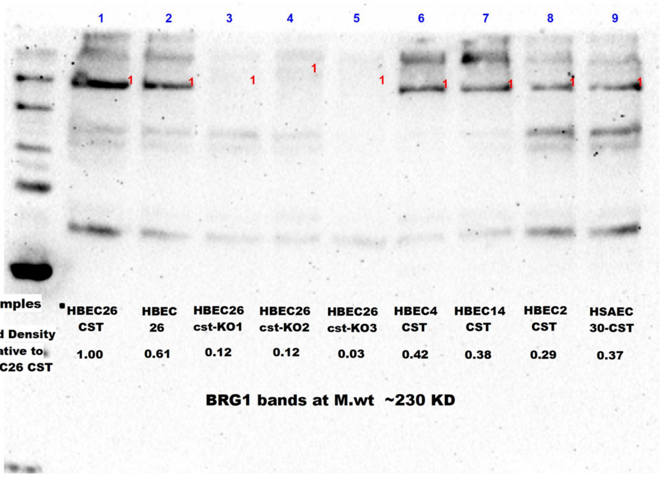

Figure 2. B BRG1(H26cst, KOs CSTs).

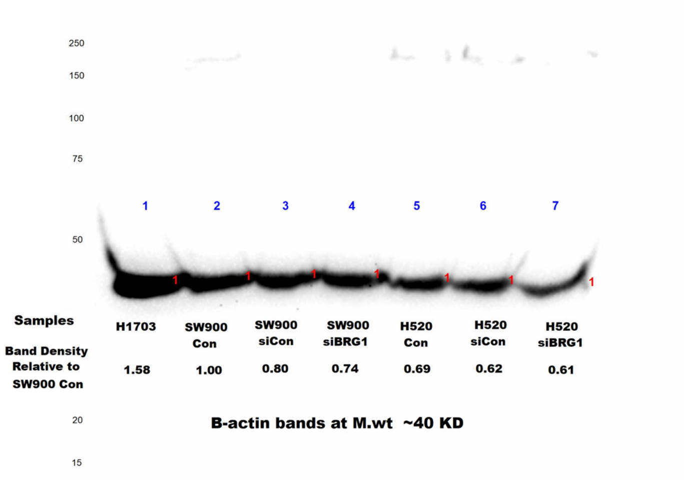

Figure S1. A B-actin(H1703 siRNA-H520 SW900).

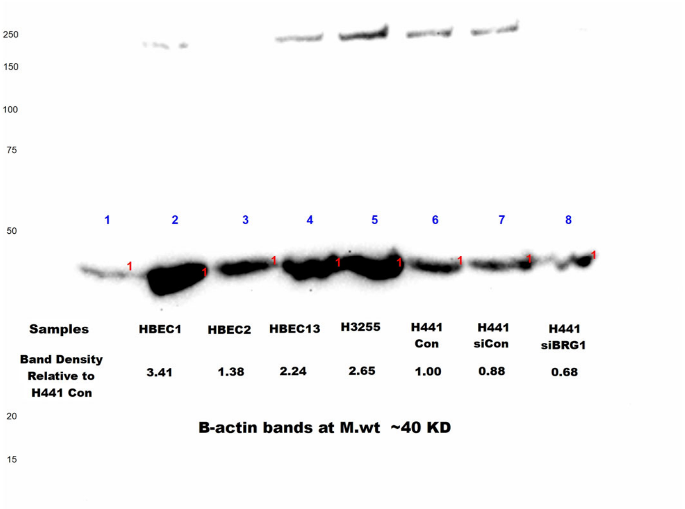

Figure S1. A B-actin(HBECs H441-siRNA).

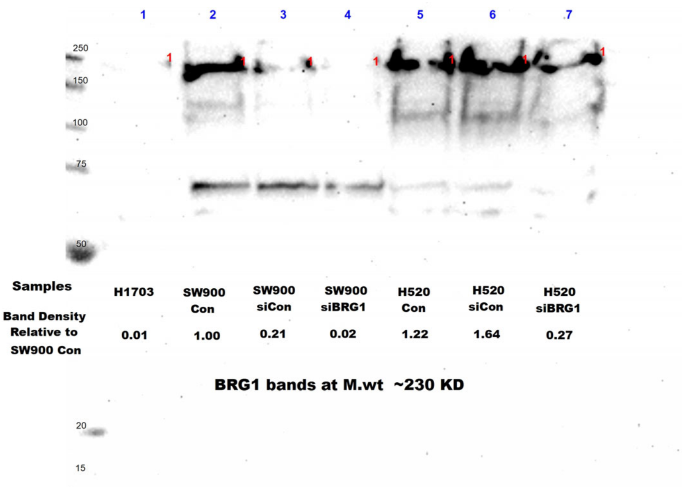

Figure S1. A BRG1(H1703 siRNA-H520 SW900).

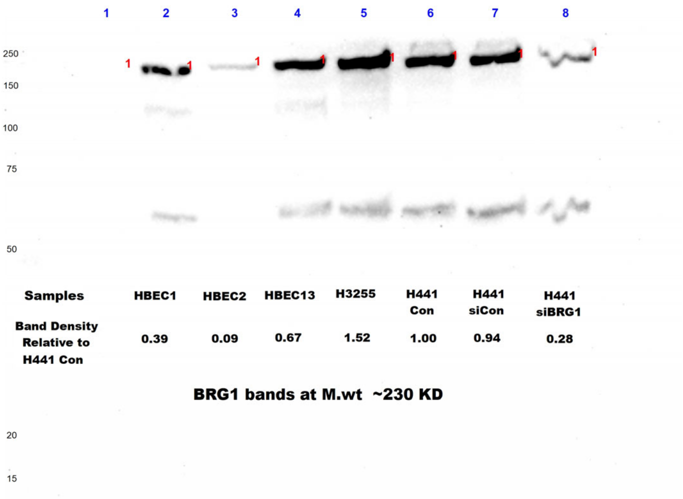

Figure S1. A BRG1(HBECs H441-siRNA).

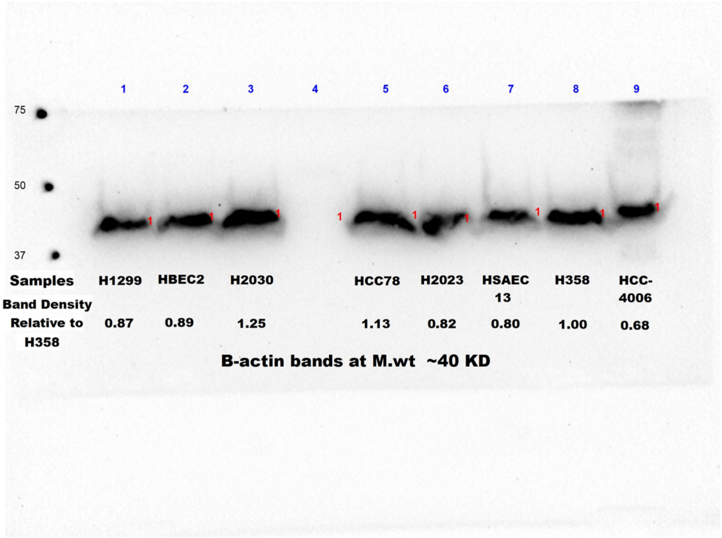

Figure 1. A 1B and othes B-actin.

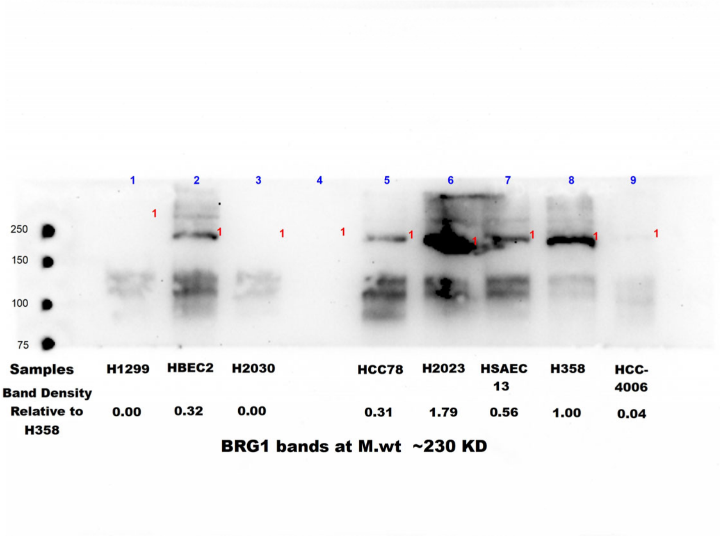

Figure 1. A 1B and othes BRG1.
